# Supplementary material for: Combining DNA and HPTLC profiles to differentiate a pain relief herb, Mallotus repandus, from plants sharing the same common name, “Kho-Khlan”
Source: PLoS One. 2022 Jun 9;17(6):e0268680. doi: 10.1371/journal.pone.0268680 (PMC9200221; doi:10.1371/journal.pone.0268680)

S1 Fig. Confirmation of CD3 (*M. repandus*) by sequencing of PCR amplicons after Bar-HRM analysis. (A) DNA alignment of the CD3 sequence with sequences of authentic *A. cocculus*, *C. caudatus* and *M. repandus*, (B) electropherogram showing partial sequence obtained from Bar-HRM amplicon. Red box presents identical nucleotide sequences among the authentic *M. repandus* sequence and CD3 sequence. Green box shows the same area between nucleotide alignment and electropherogram. "·" indicates identical nucleotide sequence. "-" presents no electropherogram result.

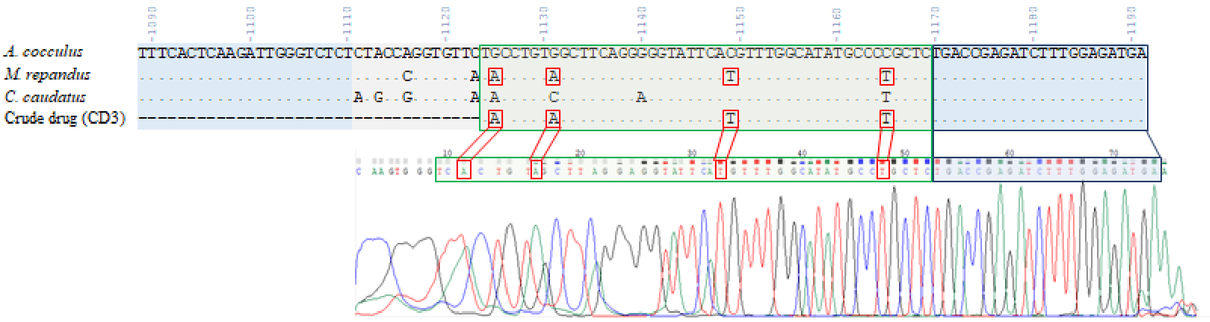

Supplement: S1 Fig — (A) DNA alignment of the CD3 sequence with sequences of authentic A. cocculus, C. caudatus and M. repandus, (B) electropherogram showing the partial sequence obtained from the Bar-HRM amplicon. The red box presents identical nucleotide sequences among the authentic M. repandus sequence and CD3 sequence. The green box shows the same area of nucleotide alignment and electropherogram. “⋅” indicates an identical nucleotide sequence. “-” indicates no electropherogram result. (PDF) [file pone.0268680.s001.pdf]
