# Supplementary material for: Combining DNA and HPTLC profiles to differentiate a pain relief herb, Mallotus repandus, from plants sharing the same common name, “Kho-Khlan”
Source: PLoS One. 2022 Jun 9;17(6):e0268680. doi: 10.1371/journal.pone.0268680 (PMC9200221; doi:10.1371/journal.pone.0268680)
Supplement: S1 Appendix — (PDF) [file pone.0268680.s002.pdf]

**S1 Appendix.** Additional primers used for DNA barcode generation used in this study.

| **Species** | **Barcode region** | **Primer name** | **Primer sequence (5′-3′)** |
|---|---|---|---|
| *Mallotus repandus* | *mat*K | *mat*K52F | GGGAGGATGGAAATCCGTTG |
| | | *mat*K1253R | GCATTTGACTCCGTACCAACA |
| | | *mat*K701F | CGCTATTGGGTGAAAGATCCCT |
| | | *mat*K1920R | CCCAGTTCCCTTCCTAGACG |
| *Croton caudatus* | *mat*K | *mat*K-390F | CGATCTATTCATTCAATATTTC |
| | | *mat*K-MALPR1 | ACAAGAAAGTCGAAGTAT |
