# Supplementary material for: Combining DNA and HPTLC profiles to differentiate a pain relief herb, Mallotus repandus, from plants sharing the same common name, “Kho-Khlan”
Source: PLoS One. 2022 Jun 9;17(6):e0268680. doi: 10.1371/journal.pone.0268680 (PMC9200221; doi:10.1371/journal.pone.0268680)
Supplement: S2 Appendix — (PDF) [file pone.0268680.s003.pdf]

S2 Appendix. Sequence alignment of four core DNA barcode regions among *A. cocculus*, *C. caudatus* and *M. repandus*.

1. Sequence alignment of *rbc*L gene

```
               ....|....| ....|....| ....|....| ....|....| ....|....|
                        10         20         30         40         50
C.caudatus     ATGTCACCAC AAACAGAGAC TAAAGCAAGT GTTGGATTCA AGGCTGGTGT
M.repandus     ATGTCACCAC AAACAGAGAC TAAAGCAAGT GTTGGATTCA AGGCTGGTGT
A.cocculus     ATGTCACCAC AAACAGAAAC TAAAGCAAGT GTTGGATTCA AAGCTGGTGT

               ....|....| ....|....| ....|....| ....|....| ....|....|
                        60         70         80         90        100
C.caudatus     TAAAGATTAT AAATTGACTT ATTATACTCC TGAGTATCAA ACAAAAGATA
M.repandus     TAAAGATTAT AAATTGACTT ATTATACTCC TGAATATGAA ACCAAAGATA
A.cocculus     TAAAGATTAC AAATTGACTT ATTATACTCC TGACTATGTA ACCAAAGATA

               ....|....| ....|....| ....|....| ....|....| ....|....|
                       110        120        130        140        150
C.caudatus     CTGATATCTT GGCAGCATTC CGAGTAACTC CTCAACCTGG AGTTCCACCT
M.repandus     CTGATATCTT GGCAGCATTC CGAGTAACTC CTCAACCCGG AGTTCCGCCT
A.cocculus     CTGATACGCT AGCAGCATTC CGAGTAACTC CTCAACCTGG AGTTCCGCCT

               ....|....| ....|....| ....|....| ....|....| ....|....|
                       160        170        180        190        200
C.caudatus     GAGGAAGCAG GAGCTGCGGT AGCAGCTGAA TCTTCTACCG GTACATGGAC
M.repandus     GAGGAAGCAG GGGCTGCGGT AGCTGCTGAA TCTTCTACCG GTACATGGAC
A.cocculus     GAAGAAGCGG GGGCTGCGGT AGCTGCCGAA TCTTCTACAG GTACATGGAC

               ....|....| ....|....| ....|....| ....|....| ....|....|
                       210        220        230        240        250
C.caudatus     AACTGTGTGG ACCGACGGGC TTACCAGTCT TGATCGTTAT AAAGGACGAT
M.repandus     AACTGTGTGG ACCGATGGGC TTACCAGTCT TGATCGTTAT AAAGGACGAT
A.cocculus     AACTGTGTGG ACCGATGGAC TTACCAGTCT TGATCGTTAC AAAGGACGAT

               ....|....| ....|....| ....|....| ....|....| ....|....|
                       260        270        280        290        300
C.caudatus     GCTACGACAT CGAGCCCGTT GCTGGAGAAG AAAATCAATA TATTGCTTAT
M.repandus     GCTACCACAT CGAGCCCGTT GCTGGAGAAG AAAATCAATT TATTGCTTAT
A.cocculus     GCTACCACAT TGAGCCCGTT GCTGGGGAAG AAAATCAATA TATTTGTTAT

               ....|....| ....|....| ....|....| ....|....| ....|....|
                       310        320        330        340        350
C.caudatus     GTAGCTTACC CTTTAGACCT TTTTGAAGAA GGTTCTGTTA CTAATATGTT
M.repandus     GTAGCTTACC CCTTAGACCT TTTTGAAGAA GGTTCTGTTA CTAATATGTT
A.cocculus     GTAGCTTACC CCTTAGACCT TTTTGAAGAA GGTTCTGTTA CTAATATGTT

               ....|....| ....|....| ....|....| ....|....| ....|....|
                       360        370        380        390        400
C.caudatus     TACTTCCATT GTGGGTAATG TATTTGGGTT CAAAGCCCTA CGTGCCCTAC
M.repandus     TACTTCTATT GTGGGTAATG TATTTGGGTT CAAAGCCCTA CGTGCCCTAC
A.cocculus     TACTTCCATT GTGGGTAATG TTTTTGGGTT CAAAGCGCTA CGCGCTCTAC
```

```
                ....|....| ....|....| ....|....| ....|....| ....|....|
                        410        420        430        440        450
C.caudatus      GTCTGGAGGA TTTGCGAATC CCTCCTGCTT ATACTAAAAC TTTCCAAGGG
M.repandus      GTCTGGAGGA TTTGCGAATC CCTACTGCTT ATGTTAAAAC TTTCCAAGGG
A.cocculus      GTCTGGAGGA TCTGCGAATT CCTACTGCTT ATATTAAAAC TTTCCAAGGC

                ....|....| ....|....| ....|....| ....|....| ....|....|
                        460        470        480        490        500
C.caudatus      CCGCCTCATG GTATCCAAGT TGAGAGAGAT AAATTGAACA AGTACGGCCG
M.repandus      CCGCCTCATG GCATCCAAGT TGAGAGAGAT AAATTGAACA AGTACGGTCG
A.cocculus      CCGCCTCATG GCATCCAAGT TGAGAGAGAT AAATTGAACA AGTATGGTCG

                ....|....| ....|....| ....|....| ....|....| ....|....|
                        510        520        530        540        550
C.caudatus      CCCCCTATTA GGTTGTACTA TTAAACCTAA ATTGGGGCTA TCCGCTAAGA
M.repandus      CCCTCTATTG GGTTGTACTA TTAAACCTAA ATTGGGGCTA TCCGCTAAGA
A.cocculus      TCCCCTATTG GGATGTACTA TTAAACCAAA ATTGGGATTA TCCGCTAAGA

                ....|....| ....|....| ....|....| ....|....| ....|....|
                        560        570        580        590        600
C.caudatus      ATTATGGTAG AGCGGTTTAT GAATGTCTTC GCGGTGGACT TGATTTTACC
M.repandus      ATTATGGTAG AGCAGTTTAT GAATGTCTAC GCGGTGGACT TGATTTTACC
A.cocculus      ACTACGGTAG AGCAGTTTAT GAATGTCTCC GCGGTGGACT TGATTTTACC

                ....|....| ....|....| ....|....| ....|....| ....|....|
                        610        620        630        640        650
C.caudatus      AAAGATGATG AAAATGTGAA CTCCCAACCA TTTATGCGTT GGAGAGACCG
M.repandus      AAAGATGATG AGAACGTGAA CTCCCAACCA TTTATGCGTT GGAGAGACCG
A.cocculus      AAGGATGATG AGAACGTGAA CTCCCAACCA TTTATGCGTT GGAGAGACCG

                ....|....| ....|....| ....|....| ....|....| ....|....|
                        660        670        680        690        700
C.caudatus      TTTCCTATTT TGTGCCGAAG CAATTTATAA AGCCCAGGCT GAAACAGGTG
M.repandus      TTTCTTATTT TGTGCCGAAG CACTTTATAA AGCACAGGCT GAAACAGGTG
A.cocculus      TTTCCTATTT TGTGCTGAAG CACTTTATAA AGCACAAGCC GAAACGGGTG

                ....|....| ....|....| ....|....| ....|....| ....|....|
                        710        720        730        740        750
C.caudatus      AAATCAAAGG ACATTATTTG AATGCTACTG CAGGTACATG TGAAGAAATG
M.repandus      AAATCAAAGG ACATTATTTG AATGCTACCG CAGGTACATG CGAAGAAATG
A.cocculus      AAATCAAAGG ACATTACTTG AATGCTACTG CAGGTACATG CGAAGAAATG

                ....|....| ....|....| ....|....| ....|....| ....|....|
                        760        770        780        790        800
C.caudatus      ATCAAAAGGG CTGTATTTGC CAGAGAATTA GGAGCTCCTA TCGTAATGCA
M.repandus      ATGAAAAGGG CTGTATTTGC CAGAGAATTA GGAGTTCCTA TCGTAATGCA
A.cocculus      ATCAAAAGGG CTGTATGTGC CAGAGAGTTG GGAGTCCCTA TTGTAATGCA

                ....|....| ....|....| ....|....| ....|....| ....|....|
                        810        820        830        840        850
C.caudatus      TGACTACCTA ACAGGGGGAT TCACCGCAAA TACTTCCTTG GCTCATTATT
```

```
M.repandus   TGATTACTTA ACAGGGGGAT TCACTGCAAA TACTAGCTTG GCTCATTATT
A.cocculus   TGACTACTTA ACGGGAGGAT TCACTGCGAA TACTTCCTTG GCTCATTATT

             ....|....| ....|....| ....|....| ....|....| ....|....|
                   860        870        880        890        900
C.caudatus   GCCGAGATAA TGGTTTACTT CTTCACATTC ACCGCGCAAT GCATGCAGTT
M.repandus   GCCGAGATAA TGGTTTACTT CTTCACATTC ACCGTGCAAT GCATGCAGTT
A.cocculus   GCCGAGATAA TGGTCTACTT CTTCACATCC ACCGCGCAAT GCATGCAGTT

             ....|....| ....|....| ....|....| ....|....| ....|....|
                   910        920        930        940        950
C.caudatus   ATTGATAGAC AGAAGAATCA TGGTATGCAT TTTCGTGTAC TAGCTAAGGC
M.repandus   ATTGATAGAC AGAAGAATCA TGGTATGCAC TTTCGTGTAC TAGCGAAGGC
A.cocculus   ATTGATAGAC AGAAGAATCA TGGTATGCAT TTCCGTGTAC TAGCTAAAGC

             ....|....| ....|....| ....|....| ....|....| ....|....|
                   960        970        980        990       1000
C.caudatus   GTTACGTATG TCTGGTGGAG ATCATATTCA CGCTGGTACC GTAGTAGGTA
M.repandus   GTTACGTATG TCTGGTGGAG ATCATATTCA CGCTGGTACC GTAGTAGGTA
A.cocculus   GTTACGTATG TCCGGTGGAG ATCATATTCA CGCTGGTACC GTAGTAGGTA

             ....|....| ....|....| ....|....| ....|....| ....|....|
                  1010       1020       1030       1040       1050
C.caudatus   AACTTGAAGG GGAAAGAGAC ATCACTTTGG GCTTTGTTGA TTTACTACGC
M.repandus   AACTTGAAGG GGAAAGAGAC ATCACTTTGG GCTTTGTGGA TTTATTGCGT
A.cocculus   AACTGGAAGG GGAAAGAGAA ATCACCCTGG GCTTTGTTGA TTTACTACGC

             ....|....| ....|....| ....|....| ....|....| ....|....|
                  1060       1070       1080       1090       1100
C.caudatus   GATGATTTTA TTGAAAAAGA CCGAAGCCGT GGTATTTATT TCACTCAAGA
M.repandus   GATGATTTTA TTGAAAAAGA TCGAAGCCGC GGTATTTATT TCACTCAAGA
A.cocculus   GATGATTTTA TTGAAAAAGA CCGCAGTCGC GGTATTTATT TCACTCAAGA

             ....|....| ....|....| ....|....| ....|....| ....|....|
                  1110       1120       1130       1140       1150
C.caudatus   TTGGGTCTCT ATGCCGGGTG TTATACCTGT CGCTTCAGGA GGTATTCACG
M.repandus   TTGGGTCTCT CTACCCGGTG TTATACCTGT AGCTTCAGGG GGTATTCATG
A.cocculus   TTGGGTCTCT CTACCAGGTG TTCTGCCTGT GGCTTCAGGG GGTATTCACG

             ....|....| ....|....| ....|....| ....|....| ....|....|
                  1160       1170       1180       1190       1200
C.caudatus   TTTGGCATAT GCCTGCTCTG ACCGAGATCT TTGGAGATGA TTCCGTACTA
M.repandus   TTTGGCATAT GCCTGCTCTG ACCGAGATCT TTGGAGATGA TTCTGTACTA
A.cocculus   TTTGGCATAT GCCCGCTCTG ACCGAGATCT TTGGAGATGA TTCCGTACTA

             ....|....| ....|....| ....|....| ....|....| ....|....|
                  1210       1220       1230       1240       1250
C.caudatus   CAATTTGGCG GGGGAACTTT AGGGCACCCT TGGGGAAATG CGCCTGGTGC
M.repandus   CAATTCGGGG GAGGAACTTT AGGACACCCT TGGGGAAATG CACCCGGTGC
A.cocculus   CAGTTCGGTG GAGGAACTTT AGGACACCCT TGGGGAAATG CACCGGGCGC

             ....|....| ....|....| ....|....| ....|....| ....|....|
```

```
                        1260       1270       1280       1290       1300
C.caudatus   CGTAGCGAAT CGAGTAGCTC TAGAAGCATG TGTACAAGCT CGTAATGAAG
M.repandus   CGTAGCTAAT CGAGTAGCTC TAGAAGCATG TGTACAAGCT CGTAATGAGG
A.cocculus   TGTAGCTAAT CGAGTAGCTC TAGAAGCGTG TGTACAAGCT CGTAATGAAG


             ....|....| ....|....| ....|....| ....|....| ....|....|
                   1310       1320       1330       1340       1350
C.caudatus   GACGTGATCT TGCTCGTCAG GGTAATGAAA TTATCCGCGA GGCTAGCAAA
M.repandus   GGCGTGATCT TGCTCGCGAG GGTAATGAAA TTATCCGCGA AGCTAGCAAA
A.cocculus   GGCGTGATCT TGCTCGTGAA GGTAATGAAA TTATCCGTGA GGCTAGCAAA


             ....|....| ....|....| ....|....| ....|....| ....|....|
                   1360       1370       1380       1390       1400
C.caudatus   TGGAGTCCGG AACTAGCTGC TGCTTGTGAA GTATGGAAGG AGATTAAATT
M.repandus   TGGAGTCCTG AACTAGCCGC TGCTTGTGAA GTATGGAAGG AAATTAAATT
A.cocculus   TGGAGCCCTG AACTAGCTGC TGCTTGTGAG GTATGGAAGG AGATCAAATT


             ....|....| ....|....| ....|...
                   1410       1420
C.caudatus   TGAATTCGAA GCAGTGGATA CTTTGTAA
M.repandus   TGAATTCCCT GCAATGGATA CTATTTGA
A.cocculus   CGAATTTGAC GCAGTGGATA CTTTGTAA
```

2. Sequence alignment of *mat*K gene

```
             ....|....| ....|....| ....|....| ....|....| ....|....|
                     10         20         30         40         50
C.caudatus   ATGTGGGAAT ACCTGAGATA TTTAGAACTA GATAGGTCTC G------AAA
M.repandus   ATGGAGGAAT ATCAAAGATA TTTAGAACTC GATAGATCTC G------AAA
A.cocculus   ATGGAAGAAT TACAAGCATA TTTCGAAATA GATAGATCTC GACAAAAAGA


             ....|....| ....|....| ....|....| ....|....| ....|....|
                     60         70         80         90        100
C.caudatus   AAAGGACTTC CTGTACCCAT TTATTTTTCG GGAGTATATT TATACATTTG
M.repandus   AAATGACTTC CTATACCCAT TTATTTTTCG GGAGTATATT TACACATTTG
A.cocculus   AAAAGACTTC CTATATCCAC TTCTTTTTCA GGAGTATATT TATGCCCTTG


             ....|....| ....|....| ....|....| ....|....| ....|....|
                    110        120        130        140        150
C.caudatus   CTCATGATCA TAATTTAAAT AGATCTATTT TGT------- --TGGAAAAT
M.repandus   CTCATGATCA CAGTTTAAAT AGATCTACTT TGT------- --TGGAAAAT
A.cocculus   CTCATGATTA TGGTTTAACT AGAGCGATTC TTTACGAACC TGTGGCAAAT


             ....|....| ....|....| ....|....| ....|....| ....|....|
                    160        170        180        190        200
C.caudatus   GTGGGTTATG ACAATAAATC TAGTTTTTTA ATTGTAAAAC GTTTAATTAC
M.repandus   GTAGGTTATG ACAATAAATC TAGTTTATTA ATTATAAAAC GTTTAATTAT
A.cocculus   TTAGGTTATG ACAATAAATC TAGTTCACTA ATTGTAAAAC GTTTAATTAC


             ....|....| ....|....| ....|....| ....|....| ....|....|
                    210        220        230        240        250
```

```
C.caudatus   TCGAATGTAT CAACAGAATC ATTTTCTTTT TTCTGCTAAT GATTCTAACC
M.repandus   TCGAATGTAT CAACAGAATC ATTTGATTAT TTCTACTAAT GATTCTAATC
A.cocculus   TCGAATGTAT CAACAGAATC ATTTGATTCT TTCTTTTAAT GATTCTAAAA

             ....|....| ....|....| ....|....| ....|....| ....|....|
                    260        270        280        290        300
C.caudatus   AAAATTCATT TTTTAGGTAC AACAAGAATT TGTATTATCA AATGATATCA
M.repandus   AAAATCCATT TTTTAGGTAC AACAAGAATT TGTATTATCA AATGCTATCA
A.cocculus   AAAATMCATT TTTGGGGTAC AAGAATCATT TTGATTCTCA AATCATATCA

             ....|....| ....|....| ....|....| ....|....| ....|....|
                    310        320        330        340        350
C.caudatus   GAGAGTTTTG CAATTATTGT GGAAATTCCA TTTTCTCTAC AATTAGTATC
M.repandus   GAGGGGTTTG CAGTTATTGT GGAAATTCCA TTTTCCTTAC GATTAGTATC
A.cocculus   GAGGGATTTG CCGTCATTGT GGAAATTCCA TTCTCGTTGC GATTAGTATC

             ....|....| ....|....| ....|....| ....|....| ....|....|
                    360        370        380        390        400
C.caudatus   TTCTTTGGAA AGGTCGGAGA CAATAAAATC TCATAAATTA CGATCAATTC
M.repandus   TTCTTTAGGA AGGTCAGAGA TAGTAAAGTC TAATAAATTA CGATCAATTC
A.cocculus   CTCCCTAGAA GGGAAAGAAA TAGCCAAATC GCATAATTTA AGATCAATTC

             ....|....| ....|....| ....|....| ....|....| ....|....|
                    410        420        430        440        450
C.caudatus   ATTCAATATT TCCTTTTTTA GAGGATAAAT TTCCACATTT AAATTATGTA
M.repandus   ATTCAATATT TCCTTTTTTA GAGGACAAAT TTCCACATTT AAATTATGTG
A.cocculus   ATTCACTATT TCCATTTTTA GAGGATCAAT TTTCACATTT AAATCATGTA

             ....|....| ....|....| ....|....| ....|....| ....|....|
                    460        470        480        490        500
C.caudatus   TCAGATGTGT TAATACCTTA CCCCATCCAT GTAGAAAAAT TAGTTCAAAT
M.repandus   TCAGATGTAT TAATACCTTA CCCTATCCAC CTAGAAAAAT GGGTTCAAAT
A.cocculus   TTAGATATAC TAATACCCTA CCCCATCCAT CTGGAACTGT TGGTTCAAAC

             ....|....| ....|....| ....|....| ....|....| ....|....|
                    510        520        530        540        550
C.caudatus   CCTTCGCTAT TGGATGAAAG ATCCCTCGTC TTTGCATTTA TTACGGCTCT
M.repandus   CCTTCGCTAT TGGGTGAAAG ATCCCTCTTC TTTGCATTTA TTACGACTCT
A.cocculus   CCTTCGCTGT TGGATACAAG ATGTCGCTTC TTTGCATTTA TTGCGATTCT

             ....|....| ....|....| ....|....| ....|....| ....|....|
                    560        570        580        590        600
C.caudatus   TTCTTCATGA GTATTGGAAT AGGAGCAGTC TTTTTATTC- --AAAAGAAA
M.repandus   TTCTTCATGA ATATTTGAAT TGGAACAATT TTATTATTC- --CAAAGCAA
A.cocculus   TTTTCTACGA GTATCATAAT TGGAATAGTC TTATTACTCA AAAAAAAAAA

             ....|....| ....|....| ....|....| ....|....| ....|....|
                    610        620        630        640        650
C.caudatus   TCTATTTTTA TTTTTACAAA AA---GTAAT CCAAGATTTT TCTTGTTCCT
M.repandus   TCTATTTCTA TTTTTACAAA AA---GTAAT CCAAGATTTT TCTTGTTCCT
A.cocculus   TCCATTTCCG TTTTTTCAAA AAAAGAAAAT CAAAGACTAT TCTTGTTCCT
```

```
              ....|....| ....|....| ....|....| ....|....| ....|....|
                     660        670        680        690        700
C.caudatus    ATATAATTCT CATGTATATG AATACGAATC AATCCTCTTT TTTCTTCGTA
M.repandus    ATATAATTCT TATGTATATG AATATGAATC CATCTTCTTT TTTCTCCGTA
A.cocculus    ATATAATTCT CATGTATATG AATGCGAATC GATATTTGTT TTTCTCCGTA


              ....|....| ....|....| ....|....| ....|....| ....|....|
                     710        720        730        740        750
C.caudatus    ACCAATCCTT TCATTTACGA TCAACATTTT CTCGAGTCCT TCTTGAACGA
M.repandus    ACCAATCCTT TCATTTACGA TCAACATTTT CTCGAGTACT TCTTGAACGA
A.cocculus    AACAATCTTT TTATTTACGA TTAACATCTT CTAGCGCCTT TCTGGAGCGA


              ....|....| ....|....| ....|....| ....|....| ....|....|
                     760        770        780        790        800
C.caudatus    ATTTTTTTCT ATAGAAAAAT AGAACATTTT GCAGAAGTCT TTGCTAATGA
M.repandus    ATTTTTTTCT ATGGAAAAAT AGAACATTTT GCGGAAGTCT TTGCTAATGA
A.cocculus    ACCCATTTCT ATGGAAAAAT GGAACATCTT GTAGTAGTTT TTCAAAATGA


              ....|....| ....|....| ....|....| ....|....| ....|....|
                     810        820        830        840        850
C.caudatus    TTTTCAGACT ATCCTAGGGT TGGTCAAAGA TCCTCTCCTG CATTATGTTA
M.repandus    TTTTCAGGCC ATCCTATGGT TATTCAAGGA TCCTTTCATG CATTATGTTA
A.cocculus    TTTTCAGTTT ATCCTATGGT TGTTCAGGGA GCCTTTTATG CATTATGTCA


              ....|....| ....|....| ....|....| ....|....| ....|....|
                     860        870        880        890        900
C.caudatus    GATATCAAGG AAAATCCATT CTGGCTTCAA AAGATGGGCT TCTTCTGATG
M.repandus    GATATCAAAG AAAATCCATT TTGGCTTCAA AAGGTGGGCC TCTTCTGATG
A.cocculus    GATATCGAGG AAAATCCATT CTGGGTTCAA AGGGGACCCC TCTTTTGATG


              ....|....| ....|....| ....|....| ....|....| ....|....|
                     910        920        930        940        950
C.caudatus    AAAAAATGGA AATATTACCT TGTCAATTTA TGTCAATGTC ATTTTTATGT
M.repandus    AAAAAATGGA AATATTTTCT TGTCAATTTT TGTCAATGTC ATTTTTATGT
A.cocculus    AATAAATGGA ACTATTACCT TGTAAATTTC TGGCAATGTA ATTTTGACTT


              ....|....| ....|....| ....|....| ....|....| ....|....|
                     960        970        980        990       1000
C.caudatus    ATGGTTTCAA CCAAAAAAGA TCTATATAAG TTCATTACCC AAGCATTCTC
M.repandus    GTGGTTTCAA CCAGAAAAGA TCTATATAAA TTCATTATCT AAGCATTCTC
A.cocculus    GTGGTCTCAA CTGGATAGGA TTTATATAAC CCAATTAGCC AATCATTACT


              ....|....| ....|....| ....|....| ....|....| ....|....|
                    1010       1020       1030       1040       1050
C.caudatus    TCAACCTTTT GGGCTATCTT TCAAATGTAC AATTAAATCC TTTGGTCGTA
M.repandus    TCAACTTTTT GGGCTATCTT TCAAATGTAC AATTCAATCC TTTGTTGGTA
A.cocculus    TCGATTTTTT GGACTATCTT TCAAGTGTAC GACTAAATAC TTTAGTAGTA


              ....|....| ....|....| ....|....| ....|....| ....|....|
                    1060       1070       1080       1090       1100
C.caudatus    CGAAGTCAAA TGTTAGAAAA TTCATTTTTA ATAGAAAAAG ATAATACTAT
M.repandus    CGGAGTCAAA TGCTAGAAAA TTCATTTATA ATAGATAAAG ATAATACTAT
A.cocculus    AGGAGTCAAA TGTTAGATAA TTCATTTATT AT------GG ATATTGCGAT
```

```
                ....|....| ....|....| ....|....| ....|....| ....|....|
                        1110       1120       1130       1140       1150
C.caudatus      GAAGAAACTT GATACAATAG TTCCAATTAT TCCTTTGATT GGATCATTAT
M.repandus      GAAGAAATTC GATACAATAG TTCCAATTAT TACTTTAATT GGGTCATTGA
A.cocculus      TAAGAGGTTC GATAGTATAG TTCCAATTAC TCCTTTGATT GGATCATTGG

                ....|....| ....|....| ....|....| ....|....| ....|....|
                        1160       1170       1180       1190       1200
C.caudatus      CAAAAATGAA ATTTTGTAAT ACAGTAGGGC ATCCCATTAG TAAATCGGCC
M.repandus      CAAAAACGAA ATTTTGTAAC GCAGTAGGAC ATCCCATTAG TAAACCGGCC
A.cocculus      CTAAAGCGAA ATTTTGTAAT GTATCAGGGC ATCCCATTAG TAAGCCGGCT

                ....|....| ....|....| ....|....| ....|....| ....|....|
                        1210       1220       1230       1240       1250
C.caudatus      TGGACTGATT CATTGGATTC TGATATTATC GACCGCTTTG TGCGTATATG
M.repandus      CGGGCTGATT CCGCGGATTC TGATATTATC GATCGATTTG TGCGTATATA
A.cocculus      CGGGCCGATT CATCAGATTC TGATATTATC GATAGATTTG GTCGAATATA

                ....|....| ....|....| ....|....| ....|....| ....|....|
                        1260       1270       1280       1290       1300
C.caudatus      CAGAAAGCTT TCTCATTATT ATAGTGGATC TTCAAAAAAA AAGAGTTTGT
M.repandus      CAGAAATCTT TCTCATTATT ATAGCGGGTC TTCAAAAAAA AAGAATTTGT
A.cocculus      TAAAAATATT TCTCATTATT ACAGCGGATC CTCAAAAAAA AAGAGTTTGT

                ....|....| ....|....| ....|....| ....|....| ....|....|
                        1310       1320       1330       1340       1350
C.caudatus      ATCGAGTAAG ATATATACTT CGACTTTCGT GTGTTAAAAC TTTGGCTCGT
M.repandus      ATCGAATAAA ATATATACTT CGATTTGCTT GTGTTAAAAC TTTGGCTCGT
A.cocculus      ATCGAATAAA GTATATACTT CGACTTTCTT GTGCTAGAAC TTTGGCTCGT

                ....|....| ....|....| ....|....| ....|....| ....|....|
                        1360       1370       1380       1390       1400
C.caudatus      AAACACAAAA GCACTGTACG CGCTTTTTTG AAAAGATTAG GTTCGGAATT
M.repandus      AAACACAAAA GTACTGTACG TGCTTTTTTG AAAAGATTAG GTTCGAAATT
A.cocculus      AAACACAAGA GTACTGTACG TTCTTTTTTG AAAAGATTCG GTTCGAAATT

                ....|....| ....|....| ....|....| ....|....| ....|....|
                        1410       1420       1430       1440       1450
C.caudatus      ATTCGAAGAA TTTTTTACGG AAGAAGAACA GATTCTTTCT TTGATCTTCC
M.repandus      ATTAGAAGAA TTTTTTATGG AGGAAGAACA GATTCTTTCT TTGATCTTCC
A.cocculus      TTTGGAAGAA TTTTTTCTGG AGCAAGGGCA AGTTCTTTCT TTGATCTTCC

                ....|....| ....|....| ....|....| ....|....| ....|....|
                        1460       1470       1480       1490       1500
C.caudatus      CGAAAGTTTC CTCTATTTCG CGCAGGTTAT ATAGAGGGCG AGTTTGGTAT
M.repandus      CAAGAGTTTC TTCTATTTCG CGCAGGTTAT ATAGAGGACG GTTTTGGTAT
A.cocculus      CCCAAGCCCC TTCTTCTTTT CGTAGGTTAT ATAGAGAGCG GATTTGGTAT

                ....|....| ....|....| ....|....| ....|....| ..
                        1510       1520       1530       1540
C.caudatus      TTGGATATCA TTTCTATCAA TGATTTGGCC AATCATGAAT AA
```

```
M.repandus   TTGGATATTA TTTCTATTAA TGATTTGGCC AATCATGAAT AA
A.cocculus   TTGGATATTA TTCGTATCAA TGATCTGGCC AATTATGAAT GA
```

3. Sequence alignment of *psb*A-*trn*H intergenic spacer

```
             ....|....| ....|....| ....|....| ....|....| ....|....|
                    10         20         30         40         50
C.caudatus   ---------- ---------- ---------- ACTAGCT-TC TGTCGAGCTC
M.repandus   ---------- ---------A TTTCCTTGGA ACTAGCT-GC TGTCGAGCTC
A.cocculus   TGAACGTAAT GCTCCCAACT TCCCTCTAGA TCTAGCTGCT GTTGAAGCTC

             ....|....| ....|....| ....|....| ....|....| ....|....|
                    60         70         80         90        100
C.caudatus   CATCTATCAA TGGATAAGAC TCTGATCTTA GTGTGTACGA GTTCTTGAAA
M.repandus   CATCGACAAA TGGATAAGAT TTCGGTCTTA GTATGCGCGA GTTCTTGAAA
A.cocculus   CATCTACAAA TGGCTAAGAT TTCGGTTTTA GTGTATACGA GTCGTTGAAA

             ....|....| ....|....| ....|....| ....|....| ....|....|
                   110        120        130        140        150
C.caudatus   ACTATAACTC TAAAAGGGGG CTATTGCTCC CTTTTTAGGG TT--------
M.repandus   ATAAAAAATA TATATTTATA TATTATATAT ATATGAAAAA TATATATGAA
A.cocculus   TTGAAGGAGC A--------- ---------- ---------- ----------

             ....|....| ....|....| ....|....| ....|....| ....|....|
                   160        170        180        190        200
C.caudatus   ---------- ---------- ---------- ---------- ----------
M.repandus   AATATATATG AAATAGAAAA ATATATATGA AATATATATG AAATGAAAAA
A.cocculus   ---------- ---------- ---------- ---------- ----------

             ....|....| ....|....| ....|....| ....|....| ....|....|
                   210        220        230        240        250
C.caudatus   ---------- ---------- ---------- --------AT AGTTTTTTTT
M.repandus   AAGGGGGGCA ATCGCCAATT TCTTGTTCTA TCAAGACAAG GGGGTTGGTA
A.cocculus   ---------- ATACCCAATT TTTTGTTCTA TCAAAGGGGC GGGTATTGCC

             ....|....| ....|....| ....|....| ....|....| ....|....|
                   260        270        280        290        300
C.caudatus   TTAGT----- --------AT TTTTTTT--- ---------- ----------
M.repandus   TTGCT----- --------CC TTTTTTTTTT A--------- -------TTT
A.cocculus   CCTTCAGTTG GGGTTAGTAG TGTTTTACTC ACATAAGTAT TTTTGACATT

             ....|....| ....|....| ....|....| ....|....| ....|....|
                   310        320        330        340        350
C.caudatus   TTCAATTTCA AATATCAAAT AA-------- --TAGATAAA GCTTTCATTT
M.repandus   ATATATTATA TATATATAAT TATATATAAT TATATATATA TGTTTAATAT
A.cocculus   TTCCATTTCT TTCTTTCAAC TTAAGAAATT CATAAAAAAA GCATTAATAC

             ....|....| ....|....| ....|....| ....|....| ....|....|
                   360        370        380        390        400
C.caudatus   TTA------- ---TGC---- ---------- ---------- ----------
M.repandus   ATA------- ---TATATGT TTAATATGTT TACTTTACAT AGTTTACTTT
```

```
A.cocculus    GGATTTTTTT TATTATTTTT TTATTATTTT T--CTTGAGT ATTATGCTTT

              ....|....| ....|....| ....|....| ....|....| ....|....|
                     410        420        430        440        450
C.caudatus    ---------- ---------- ---------- ---------- ----------
M.repandus    ACATAAACAT AAAAAATAAA AAGAAAAAGT TTATTTAAAG ATTAAAGAAA
A.cocculus    GTTTCTGTAC TAATAGTACT A--------- ---------- -----ATATA

              ....|....| ....|....| ....|....| ....|....| ....|....|
                     460        470        480        490        500
C.caudatus    ---------- ---------- ---------- ---------- ----------
M.repandus    AAAGAACAAA AACAAAGAAT CTTTTAGATT TTAGAAGATT TTAGAAAAAG
A.cocculus    TAATAAC--- ------ATAT ATGTT----- ---------- ----------

              ....|....| ....|....| ....|....| ....|....| ....|....|
                     510        520        530        540        550
C.caudatus    ---------- ---------- ---------- ---------- ----------
M.repandus    AATCTTTTTA TAGGTTGTTT GGGGAGGATT ATATATATAT TATGTACTAA
A.cocculus    ---ATTTATA TGATATGTTT CCTGACATCT TTTTCTTTCT A--------A

              ....|....| ....|....| ....|....| ....|....| ....|....|
                     560        570        580        590        600
C.caudatus    ---------- ---------- ---------- ---------- ----------
M.repandus    TTTTTAATTG AGTATATGTT CTTCTCGATC GTTTTTTTAA GATTGAAAAA
A.cocculus    TTCGAAAGTT T--TTTTATT ATTTTTTTGA GTTTATTAGA AACTCAAATA

              ....|....| ....|....| ....|....| ....|....| ....|....|
                     610        620        630        640        650
C.caudatus    ---------- ---------- ---------C TTTGATATTT TTGTAGTTGT
M.repandus    AAAAAAGATA TAAAAGATGA AGTTTGTATT TTTATGTCTT TTGTAGTTGT
A.cocculus    GAAACTTAAA TTAAAAAAAA AGGCATTTTA ATTACAATAT CTTTAT-TTC

              ....|....| ....|....| ....|....| ....|....| ....|....|
                     660        670        680        690        700
C.caudatus    ATTTTTTTGT TGTATTTGTA TTTTATAAGT TATAAGAAAG AAAAAATAGA
M.repandus    ATTTTATTCT TGTATTTGTA TCTTGTAAGA TCGTA----- ----------
A.cocculus    AGTATATTCT AAAAAGACGC ATAAGAAAGA CTTAAAGATG AAAGACTTCA

              ....|....| ....|....| ....|....| ....|....| ....|....|
                     710        720        730        740        750
C.caudatus    ATTTTAATAT TAAATCCTAA GGATTTAATA CTAGAAATTG AAATTCTTGT
M.repandus    ---------T AAGAAATTGA AGTTTGAATG CTAGAAAGTG AAATTCTTGT
A.cocculus    AGATATAAAA AAACTGAATT GAATAAAATA CTAGAAATTA AACGTTTTTT

              ....|....| ....|....| ....|....| ....|....| ....|....|
                     760        770        780        790        800
C.caudatus    ACCTTCTAAA AAAAAAAATT ATTTCATTTT ATAATAATCT TAATCTAATT
M.repandus    ACCTTCTAAT ATTTAGTCGA ATTGAAATTT AAAAG----- ----------
A.cocculus    GGG------- ---------- ---------- ---------- ----------

              ....|....| ....|....| ....|....| ....|....| ....|....|
                     810        820        830        840        850
```

```
C.caudatus   TTGAAATTTT GAAATTGAAA TAAAGTAGAA TATTGTAATA TTCTATTACG
M.repandus   -------TTG AAAAGTAGAG TATACTATAT ATATATACTA TTCTATATAT
A.cocculus   ---------- GAAAGTG--- ---------- ---------- ------CCGG

             ....|....| ....|....| ....|....| ....|....| ....|....
                    860        870        880        890
C.caudatus   GGCGGATGTA CCCAAGGGGA TTAAGGCAGG GGATTTGTGA A--------
M.repandus   ATATACTATA AAGTATAGCA TATAGTTTAG GGGCGGAT-- ---------
A.cocculus   GGCGGATGTA GCCAAGTGGA TTAAGGCAGT GGATTTGTGA ATCCACCCA
```

4. Sequence alignment of ITS region

```
             ....|....| ....|....| ....|....| ....|....| ....|....|
                     10         20         30         40         50
C.caudatus   TCGAAACCTG CACAGCAGAA CGACTCGTGA ACAAGTAAGA TAACACTCGG
M.repandus   TCGAAACCTG CTCTGCAGAA CGACCCGCGA ACACGTTCAC AATACATGCG
A.cocculus   TCGAAACCTG CAAAGCAGAA AGA------- ---------- ----------

             ....|....| ....|....| ....|....| ....|....| ....|....|
                     60         70         80         90        100
C.caudatus   GTGACCTCTG GGGCCTTCGG GCGCCATTGT GAACCTACAA GGCTGAGTCG
M.repandus   GCGGAGCGGG GGGGCTTGTG CTCCCCGG-- -ATCCACCGA TGCTGACGGG
A.cocculus   ---------- ---------- ---------- ---------- ------CCCG

             ....|....| ....|....| ....|....| ....|....| ....|....|
                    110        120        130        140        150
C.caudatus   TGCCATGTCG GGCTAGGCTT CGGCCTGCTT CACATGCACT GCTCAGTCTA
M.repandus   ACGGATGGCG GGCT------ -TCGCTCATA ACCATCCGCT CGGAGGCGCA
A.cocculus   TGAATTGTTG ACAC-----A ATCTCTCTTC AACTCGGGCT ACCA------

             ....|....| ....|....| ....|....| ....|....| ....|....|
                    160        170        180        190        200
C.caudatus   ACAACCAACC CCGGCGCAAG ACGCGCCAAG GAAAACAAAA AATGAAAGAG
M.repandus   ATAACCAACC CCGGCGCAGG TAGCGCCAAG GAATACGAAC CGAAAAGAGC
A.cocculus   -CGGCCACCG AGTTCGGTGG CCTTGCTAGT CCCGATCGAA CAACAAACCC

             ....|....| ....|....| ....|....| ....|....| ....|....|
                    210        220        230        240        250
C.caudatus   AAGGCGCGTC TGACCGGTCC -CGG---CTT CGGGATCTCG GAAAGAATGT
M.repandus   ACGCTGC--- -CACAGTTCC -CGGAAACGG CGGACCAGGG GCACGCGTCG
A.cocculus   CCGGCGCGGT TCGCGCCAAG GAAAATCTAA ATTGATGGTA CGCGGATCGC

             ....|....| ....|....| ....|....| ....|....| ....|....|
                    260        270        280        290        300
C.caudatus   GTCCTCTTTT CTTAAAAAAA CGACTCTCGG CAACGGATAT CTCGGCTCTC
M.repandus   TACTCTTTCA AAAATCATAA CGACTCTCGG CAACGGATAT CTCGGCTCTC
A.cocculus   GTTCCACTCT TATTCTTGAA CGACTCTCGG CAACGGATAT CTCGGCTCTC

             ....|....| ....|....| ....|....| ....|....| ....|....|
                    310        320        330        340        350
```

```
C.caudatus   GCATCGATGA AGAACGTAGC GAAATGCGAT ACTTGGTGTG AATTGCAGAA
M.repandus   GCATCGATGA AGAACGCAGC AAAATGCGAT ACTTGGTGTG AATTGCAGAA
A.cocculus   GCATCGATGA AGAACGTAGC GAAATGCGAT ACTTGGTGTG AATTGCAGAA

             ....|....| ....|....| ....|....| ....|....| ....|....|
                   360        370        380        390        400
C.caudatus   TCCCGCGAAC CATCGAGTTT TTGAACGCAA GTTGCGCCCA AAGCCTTTCG
M.repandus   TCCCGTGAAT CATCGAGTTT TTGAACGCAA GTTGCGCCCG AAGCCTTTCG
A.cocculus   TCCCGTGAAC CATCGAGTTT TTGAACGCAA GTTGCGCCCG AGGCCATCAG

             ....|....| ....|....| ....|....| ....|....| ....|....|
                   410        420        430        440        450
C.caudatus   GCCGAGGGCA CGTCTGCCTG GGTGTCACGC AACATCGCTC CCAACCCATT
M.repandus   GCCAAGGGCA CGTCTGCCTG GGTGTCACGC AACCGTTGCT CCCAACCCTT
A.cocculus   GTCGAGGGCA CGTCTGCCTG GGCGTCACGC AATGCGCCAC TCCCACCCCC

             ....|....| ....|....| ....|....| ....|....| ....|....|
                   460        470        480        490        500
C.caudatus   AGGGTGCGGA ATATGGCCTC CCGTGC---- ---------- ------GATT
M.repandus   TCGACGCGTY GAGGGGCACT GGGGGCGGAT GCTGGCCTCC CGCGCGCTGC
A.cocculus   TGGGGCACGG AGCGAACATT GGCCTC---- ---------C CGCG--AACA

             ....|....| ....|....| ....|....| ....|....| ....|....|
                   510        520        530        540        550
C.caudatus   CCCTCTTGCG GTTGGCCGAA AAGAATGGTC CTCGGCTGCG AATGCCGCGA
M.repandus   GAGGCGTGCG GTTGGCCCAA AAGCCGAGTT CCGGGCGAAG GTTGCCACGA
A.cocculus   CCCGTGCACG GTTGGCCGAA AATCTGGCCC TTGGCGGCTC GACGACACGA

             ....|....| ....|....| ....|....| ....|....| ....|....|
                   560        570        580        590        600
C.caudatus   CAATCGGTGG TTGTAAGACC CTCGGA-CAC AG---TCGTG CGC-A----C
M.repandus   CAATCGGTGG TTGTAAGACC CTCTGA-AAC AG---TCGTG CGG-A----Y
A.cocculus   TCAATGGTGG TTGAAGAGAC AATACCTCGC CGCAATTGGA CGACGTGATC

             ....|....| ....|....| ....|....| ....|....| ....|....|
                   610        620        630        640        650
C.caudatus   GTACGCCTTC GGATAACGAG ACCCCTTTGC GTCCGCGTCC TTCGCGGCAC
M.repandus   AATCCTACGC CGTGAGGGAC CCCGAGACCC CGATGCAGCC TGAACGGCAT
A.cocculus   GAAGGGCAGC CGAGAAAGGG GCAAG--CGA ACCCTCGAAG TTAACACAAT

             ....|....

C.caudatus   GCTCACACC
M.repandus   GCTCCAATC
A.cocculus   ---------
```
