# Supplementary material for: Combining DNA and HPTLC profiles to differentiate a pain relief herb, Mallotus repandus, from plants sharing the same common name, “Kho-Khlan”
Source: PLoS One. 2022 Jun 9;17(6):e0268680. doi: 10.1371/journal.pone.0268680 (PMC9200221; doi:10.1371/journal.pone.0268680)
Supplement: S3 Appendix — (PDF) [file pone.0268680.s004.pdf]

S3 Appendix. Melting temperatures of PCR amplicons and cluster groups generated by various DNA concentrations.

**Species**	**DNA concentration ($\times 10^{-9}$ g)**	**Cluster**	**Melting temp**	**% Confidence**
A. cocculus	10	1	82.0	95.3
	1	1	82.0	95.5
	0.1	1	82.0	99.4
	0.01	1	82.0	98.9
	0.001	2	81.9	96.6
	0.0001	2	81.7	99.8
C. caudatus	10	1	80.9	98.1
	1	1	80.9	98.8
	0.1	1	80.9	98.9
	0.01	1	80.9	89.9
	0.001	2	80.7	98.3
	0.0001	2	80.7	96.9
M. repandus	10	1	80.0	98.0
	1	1	80.0	98.8
	0.1	1	80.0	98.9
	0.01	1	80.0	89.9
	0.001	2	79.9	96.8
	0.0001	2	79.9	98.6
