# Supplementary material for: Combining DNA and HPTLC profiles to differentiate a pain relief herb, Mallotus repandus, from plants sharing the same common name, “Kho-Khlan”
Source: PLoS One. 2022 Jun 9;17(6):e0268680. doi: 10.1371/journal.pone.0268680 (PMC9200221; doi:10.1371/journal.pone.0268680)
Supplement: S1 Raw images — (PDF) [file pone.0268680.s005.pdf]

Combining DNA and HPTLC profiles to differentiate a pain relief herb, *Mallotus repandus*, from plants sharing the same common name, "Kho-Khlan"

Kannika Thongkhao[1,#a], Chayapol Tungphatthong[1], Vipawee Pichetkun[1], Suthathip Gaewtongliam[1], Worakorn Wicatcharakornkul[1] and Suchada Sukrong[1*]

[1]Center of Excellence in DNA Barcoding of Thai Medicinal Plants, Department of Pharmacognosy and Pharmaceutical Botany, Faculty of Pharmaceutical Sciences Chulalongkorn University, Bangkok, Thailand

[#a]Current Address: School of Languages and General Education, Walailak University, Nakhon Si Thammarat, Thailand

[*]Corresponding Author
Email: suchada.su@chula.ac.th (SS)

Original image of Fig. 2 and Fig. 3.

White light

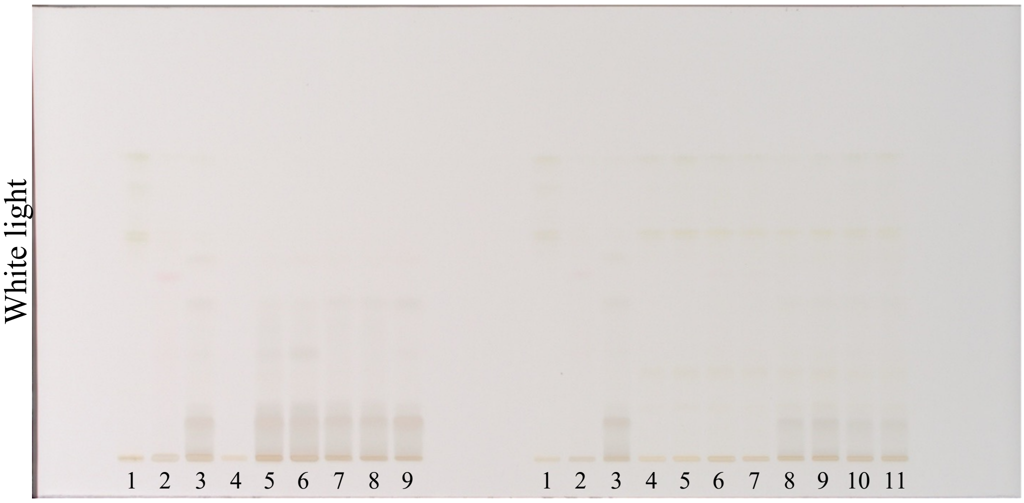

Fig. 2 Fig. 3

254 nm

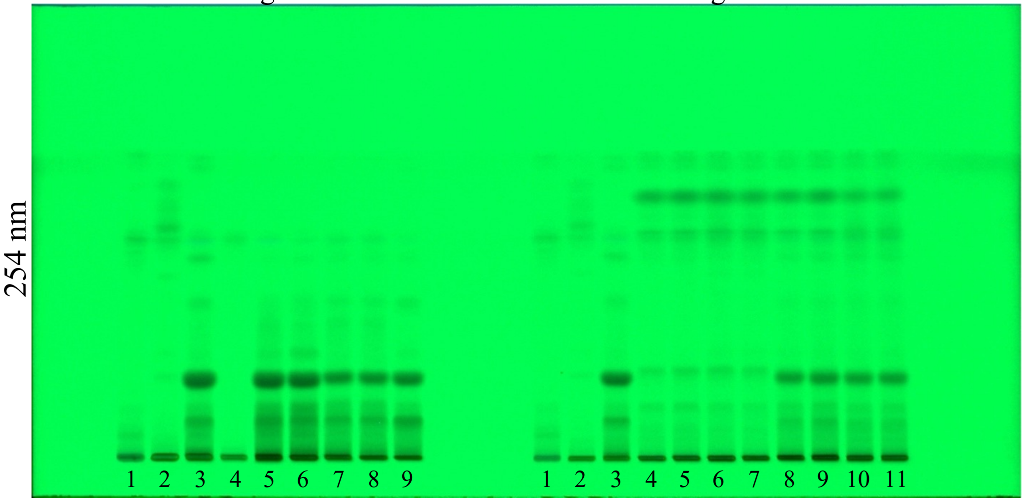

Fig. 2 Fig. 3

366 nm

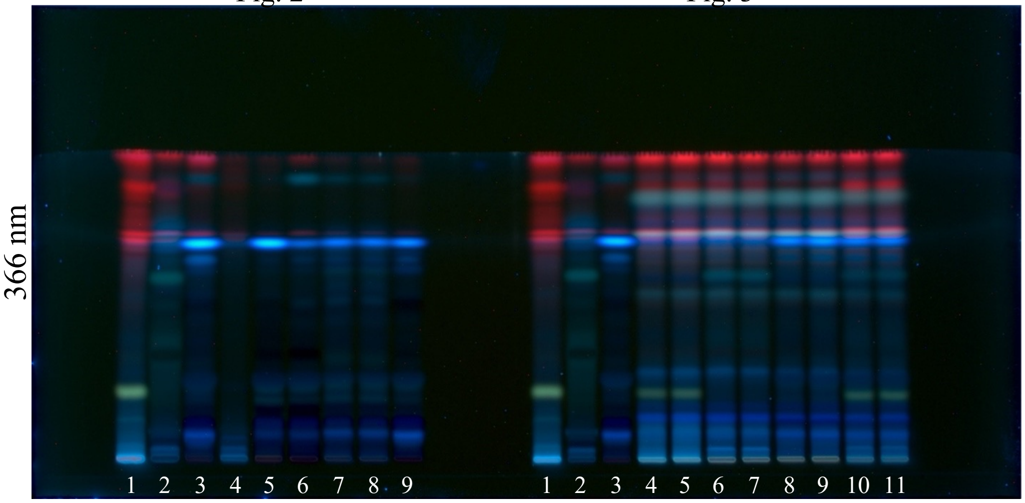

Fig. 2 Fig. 3

Stationary Phase : HPTLC Silica gel 60 F_{254} plates 20 × 10 cm
Mobile Phase : Toluene : Acetone : Formic acid (5:4:0.5, v/v/v)

Fig. 2

1 : *A. cocculus* 2 : C. caudatus
3 : *M. repandus* 4 : CD1
5 : CD2 6 : CD3
7 : CD4 8 : CD5
9 : CD6

Fig. 3

1 : *A. cocculus* ***2*** : C. caudatus
3 : *M. repandus* **4–5** : F-A
6–7 : F-C **8–9** : F-M
10–11 : F-ACM
