# Supplementary material for: Combining DNA and HPTLC profiles to differentiate a pain relief herb, Mallotus repandus, from plants sharing the same common name, “Kho-Khlan”
Source: PLoS One. 2022 Jun 9;17(6):e0268680. doi: 10.1371/journal.pone.0268680 (PMC9200221; doi:10.1371/journal.pone.0268680)
Supplement: Attachment — Submitted filename: Response to Reviewers.docx [file pone.0268680.s006.docx]

We appreciate the editor and the reviewers for the constructive comments. Each comment has been carefully considered point by point and responded. Responses to the reviewers and changes in the revised manuscript are as following:

**A point-by-point response to the comments**

**Editor comments**

**Comment** 1: Thank you for submitting your manuscript to PLOS ONE. After careful consideration, we feel that it has merit but does not fully meet PLOS ONE’s publication criteria as it currently stands. Therefore, we invite you to submit a revised version of the manuscript that addresses the points raised during the review process.

**Response:** Thank you editor for providing us a change to improve the manuscript. You encouraged us to revise as best as we can to make the manuscript high quality to publish in PLOS ONE.

**Comment 2**: Introduction should be more focused.

**Response:** The introduction section was revised to be more focused as suggestion from editor and reviewer #1 and #2. Please read our responses to “Comment 4 of the reviewer #1” and “Comment 1 of the reviewer #2”.

**Comment 3**: Additional clarification related to material and methodology used are needed.

**Response:** The manuscript has been revised. Additional clarification related to material and methodology used in the study has been added in the “Introduction” and “Materials and Methods” sections.

**“Introduction” Section**

**Line 84-97 and Line 100-113** were added to the Introduction Section to provide more information to the HPTLC and Bar-HRM methods, respectively, as following:

**Line 84-97** **of the revised manuscript:** “Thin-layer chromatography (TLC) and high-performance TLC (HPTLC), which are recommended in the herbal pharmacopoeias of many countries, including Thailand, are reliable methods for phytochemical constituent examination; however, the methods require a target compound as a standard reference [17, 18]. HPTLC, a sophisticated form of TLC, provides good separation efficiency due to the higher quality of its separation plate. HPTLC exhibits higher accuracy, reproducibility, and ability to document the results than TLC [18]. Therefore, this method has been used to determine the phytochemical profile of herbal species. However, uncertain results may occur due to environmental factors that affect the chemical composition of herbal species and biological activities of the substances [19]. In recent years, a molecular approach called the DNA barcoding technique has gained demand in species identification because it is an accurate, cost-effective and reliable tool for species identification. The DNA barcoding method provides species-level information, and small amounts of samples are needed for the identification process [19].”

**Line 100-113 of the revised manuscript:** Bar-HRM, a sequencing free method, detects signal alteration during the dissociation of double-stranded DNA into single stranded DNA. Each plant species can be differentiated by their individual melting temperature (T_m_) which correlated to their nucleotide sequences in the target region [23]. Bar-HRM analysis exhibits fast, cost-effective and reliable method, moreover, small amount of sample is required for species identification. However, Bar-HRM primer design is challenging when the target sequence has high variation rates across the target amplicon and the Bar-HRM analysis has limited when low quality of DNA template is used [24]. As mentioned above, each identification method has advantages and limitations, therefore, an integrative approach is proposed to differentiate substitutions and adulterants of herbal species [19, 25]. Combined methods can be applied to prevent the use of incorrect herbal species and support the quality of herbal materials to meet international standards [19].

**“Materials and Methods” Section** The section has been revised.

**Original:**

**Line 121-123 of the original manuscript:** Fresh leaves of *A. cocculus* (n=8), *C. caudatus* (n=8) and *M. repandus* (n=8) were collected from various locations across Thailand for the DNA barcoding experiment (Table 1).

**Revision:**

**Line 121-123 of the revised manuscript:** Fresh leaves and stems of *A. cocculus* (n=8), *C. caudatus* (n=8) and *M. repandus* (n=8) were collected from various locations across Thailand (Table 1). These collections are legally permitted.

**More information was added in the revised manuscript.**

**Line 129-130 of the revised manuscript:** All experiments were performed in accordance with relevant guidelines and regulations.

**Comment 4:** Table 2 should be reorganized as suggested by Reviewer #1.

**Response:** Table 2 has been reorganized as suggested by Reviewer #1. Selected sequencing primers were removed to Appendix S1.

**Original Table 2:** **Primers for DNA barcode generation and Bar-HRM analysis.**

| **Barcode region** | **Primer name** | **Primer sequence (5′-3′)** | **References** |
| --- | --- | --- | --- |
| **DNA barcode generation** | |  |  |
| *rbc*L | *rbc*L_aF | ATGTCACCACAAACAGAGACTAAAGC | Levin et al., 2003 |
|  | *rbc*L_aR | GTAAAATCAAGTCCACCR(G/A)CG | Kress & Erickson, 2009 |
|  | *rbc*L-Uro_569F | ATGAATGTCTTCGCGGTGGAC | This study |
|  | *rbc*L-R23 | TTTTAGTAAAAGATTGGGCCG | Ohi-Toma et al., 2006 |
| *mat*K | *mat*K-390F | CGATCTATTCATTCAATATTTC | Cuénoud et al. 2002 |
|  | *trn*K72F | TCCGAGGTATCTACTCTTTT | This study |
|  | *mat*K52F | GGGAGGATGGAAATCCGTTG | This study |
|  | *mat*K701F | CGCTATTGGGTGAAAGATCCCT | This study |
|  | *trn*K-3914F | TGGGTTGCTAACTCAATGG | Johnson et al., 1994 |
|  | *mat*K-aF | CTATATCCACTTATCTTTCAGGAGT | Kato et al., 1999 |
|  | *mat*K-MALPR1 | ACAAGAAAGTCGAAGTAT | Dunning & Savolainen, 2010 |
|  | *trn*K1895R | CGCTCAATCCTTACTCGTAT | This study |
|  | *mat*K1253R | GCATTTGACTCCGTACCAACA | This study |
|  | *mat*K1920R | CCCAGTTCCCTTCCTAGACG | This study |
|  | *mat*K-8R | AAAGTTCTAGCACAAGAAAGTCGA | Kato et al., 1999 |
|  | *trn*K-2R | AACTAGTCGGATGGAGTAG | Johnson et al., 1994 |
| *trn*H-*psb*A | *psb*A_*trn*HF | GTTATGCATGAACGTAATGCTC | Sang et al., 1997 |
|  | *psb*A-*trn*HR | CGCGCATGGTGGATTCACAATC | Sang et al., 1997 |
| ITS | ITS1 | TCCGTAGGTGAACCTGCGG | White et al., 1990 |
|  | ITS4 | TCCTCCGCTTATTGATATGC | White et al., 1990 |
|  | ITS5 | GGAAGTAAAAGTCGTAACAAGG | Bellemain et al., 2010 |
| **Bar-HRM analysis** | |  |  |
| *rbc*L | KK-rbcL-HRM-F | TTTCACTCAAGATTGGGTCTCT | This study |
|  | KK-rbcL-HRM-R | TCATCTCCAAAGATCTCGGTCA | This study |

**Revised Table 2: Primers for DNA barcode generation and Bar-HRM analysis.**

| **Barcode region** | **Primer name** | | **Primer sequence (5′-3′)** | **References** |
| --- | --- | --- | --- | --- |
| **DNA barcode generation** | | |  |  |
| *rbc*L | *rbc*L_aF | | ATGTCACCACAAACAGAGACTAAAGC | Levin et al., 2003 |
|  | *rbc*L-R23 | | TTTTAGTAAAAGATTGGGCCG | Ohi-Toma et al., 2006 |
| *mat*K | *trn*K-3914F | | TGGGTTGCTAACTCAATGG | Johnson et al., 1994 |
|  | *trn*K-2R | | AACTAGTCGGATGGAGTAG | Johnson et al., 1994 |
| *trn*H-*psb*A | *psb*A_*trn*HF | | GTTATGCATGAACGTAATGCTC | Sang et al., 1997 |
|  | *psb*A-*trn*HR | | CGCGCATGGTGGATTCACAATC | Sang et al., 1997 |
| ITS | ITS1 | | TCCGTAGGTGAACCTGCGG | White et al., 1990 |
|  | ITS4 | | TCCTCCGCTTATTGATATGC | White et al., 1990 |
| **Bar-HRM primers** | |  |  |  |
| *rbc*L | KK-rbcL-HRM-F | | TTTCACTCAAGATTGGGTCTCT | This study |
|  | KK-rbcL-HRM-R | | TCATCTCCAAAGATCTCGGTCA | This study |

**Appendix S1.** Additional primers used for DNA barcode generation used in this study.

| **Species** | **Barcode region** | **Primer name** | **Primer sequence (5′-3′)** |
| --- | --- | --- | --- |
| *Mallotus repandus* | *mat*K | *mat*K52F | GGGAGGATGGAAATCCGTTG |
|  |  | *mat*K1253R | GCATTTGACTCCGTACCAACA |
|  |  | *mat*K701F | CGCTATTGGGTGAAAGATCCCT |
|  |  | *mat*K1920R | CCCAGTTCCCTTCCTAGACG |
| *Croton caudatus* | *mat*K | *mat*K-390F | CGATCTATTCATTCAATATTTC |
|  |  | *mat*K-MALPR1 | ACAAGAAAGTCGAAGTAT |

**Comment 5:** In Figure 3 some data are missing.

**Response:** Thank you editor for asking. The authors repeated the HPTLC experiments in order to see the missing species-specific band of *C. caudatus*. We adjusted the mobile phase from toluene:acetone:formic acid mixture (5:3:0.5, v/v/v) into toluene:acetone:formic acid mixture (5:4:0.5, v/v/v) . This adjustment of mobile phase system affects the Rf in the HPTLC result therefore, the HPTLC result was rewrite. The original Fig. 2 and Fig. 3 were replaced by the revised Fig. 2 and the revised Fig. 3, respectively. The revised figures are shown below.

**Original Fig. 2: High-performance thin-layer chromatography (HPTLC) chromatogram of ethanolic extracts under UV 366 nm.**

Track 1: *A. cocculus*, track 2: *C. caudatus*, track 3: *M. repandus*, tracks 4–9: crude drugs CD1-CD6. A toluene:acetone:formic acid mixture (5:3:0.5, v/v/v) was used as the mobile phase. White arrows indicate the characteristic bands of each authentic plant species.

**Revised** **Fig. 2.** **High-performance thin-layer chromatography (HPTLC) chromatogram of ethanolic extracts under UV at 366 nm.**

Track 1: *A. cocculus*, track 2: *C. caudatus*, track 3: *M. repandus*, tracks 4–9: crude drugs CD1-CD6. A toluene:acetone:formic acid mixture (5:4:0.5, v/v/v) was used as the mobile phase. White arrows indicate the characteristic bands of each authentic plant species.

**Original Fig. 3. HPTLC chromatograms of ethanolic extracts of authentic species and four laboratory-made YPSKK formulae under UV 366 nm.** Track 1: *A. cocculus*, track 2: *C. caudatus*, track 3: *M. repandus*, tracks 4–5: F-A, tracks 6–7: F-C, tracks 8–9: F-M and tracks 10–11: F-ACM. A toluene:acetone:formic acid (5:3:0.5, v/v/v) mixture was used as the mobile phase. White arrows indicate the characteristic bands of each plant species.

**Revised Fig. 3. HPTLC chromatograms of ethanolic extracts of authentic species and four laboratory-made YPSKK formulae under UV at 366 nm.**

Track 1: *A. cocculus*, track 2: *C. caudatus*, track 3: *M. repandus*, tracks 4–5: F-A, tracks 6–7: F-C, tracks 8–9: F-M and tracks 10–11: F-ACM. A toluene:acetone:formic acid (5:4:0.5, v/v/v) mixture was used as the mobile phase. White arrows indicate the characteristic bands of each plant species.

**Additional rewrite after changing of mobile phase in HPTLC experiments.**

**Line 167 of the original manuscript:**

Toluene:acetone:formic acid (5:-:0.5, v/v/v) was used as the mobile phase

**Line 167 of the revised manuscript:** Toluene:acetone:formic acid (5:4:0.5, v/v/v) was used as the mobile phase.

**The HPTLC results were revised as following.**

**Original sentences:**

**Line 229-230** **of the original manuscript:** “Species-specific bands were obtained for authentic *A. cocculus* (Rf = 0.18), *C. caudatus* (Rf = 0.08 and 0.55) and *M. repandus* species (Rf = 0.05, 0.20, 0.63 and 0.68).”

**Line 232-237 of the original manuscript:** “A bright blue band at Rf = 0.68 for *M. repandus* was found in CD2-CD6. The blue band at Rf = 0.05 was present in CD2-CD5, while the band at Rf = 0.20 was present in CD2 and CD3. The crude drug CD1 showed an ambiguous HPTLC pattern with a faint band at Rf = 0.08. No distinct bands of *A. cocculus* (Rf = 0.18) or *C. caudatus* (Rf = 0.55) were detected in any of the crude drug samples (Fig 2).”

**Revised sentences:**

**Line 245-246 of the revised manuscript:** “Species-specific bands were obtained from authentic *A. cocculus* (Rf = 0.22), *C. caudatus* (Rf = 0.02 and 0.60) and *M. repandus* species (Rf = 0.08, 0.26, 0.68 and 0.72).”

**Line 248-253 of the revised manuscript:** “A bright blue band at Rf = 0.72 for *M. repandus* was found in CD2-CD6. The blue band at Rf = 0.08 was present in CD2-CD6, while the band at Rf = 0.26 was present in CD2-CD6. The crude drug CD1 showed an ambiguous HPTLC pattern with a faint band at Rf = 0.02. No distinct bands of *A. cocculus* (Rf = 0.22) or *C. caudatus* (Rf = 0.60) were detected in any of the crude drug samples (Fig 2).”

**Comment 6:** The authors should considerably widen the Discussion section towards providing scientific and empirical justification of the presented methodologies for non-closely related species.

**Response:** The manuscript has been revised by widen the Discussion section as recommended by the editor. The authors added a paragraph according to the scientific and empirical justification of the presented methodologies for non-closely related species in the discussion section as following.

**Line 353-364 of the revised manuscript:** “Recently, a number of reports have been published on the successful application of Bar-HRM and HPTLC analysis for the identification of related and nonclosely related species in herbal medicines. In 2018, Dual et al. applied the Bar-HRM method to identify Rhizoma Paridis and its common adulterants [28]. *Acanthus ebracteatus* Vahl, *Andrographis paniculate* (Burm.f.) Nees and *Rhinacanthus nasutus* (L.) Kurz were successfully discriminated by Bar-HRM analysis [29]. Moreover, Bar-HRM was applied to differentiate the poisonous plant *U. siamensis* from the edible vegetables *M. suavis* and *S. androgynus* for consumer safety purposes [24]. The HPTLC fingerprint revealed different phytochemical profiles between two nonrelated species, *Cyanthillium cinereum* (L.) H. Rob. (a smoking cessation herb) and its adulterant, *Emilia sonchifolia* (L.) DC. [19]. Combining HPTLC with the DNA barcode technique was previously reported for the identification of herbal raw materials such as *Aristolochia* species [30].”

**Comment 7:** The manuscript would greatly benefit if being proofread by a native English speaker.

**Response:** For the revised version, the manuscript has been proofread and edited by the American Journal Experts (AJE) service in order to improve the language. The AJE certificated was attached.

**Reviewer #1:**

The research article entitled “Combining DNA and HPTLC profiles to differentiate a pain relief herb, *Mallotus repandus*, from plants sharing the same common name (“Kho-Khlan”), *Anamirta cocculus* and *Croton caudatus*” highlights the use of HPTLC and BAR-HRM to distinguish the study plants. The study is potentially interesting, it has to be improved a lot before it is suitable for publication.

**Comments** **1**: One of the foremost concerns is that though the article is well written, still the proficiency of English is lacking in the article and it needs to improved.

**Response:** Thank you reviewer for your suggestions. The authors apologize for the unproficiency of English in the manuscript.

For the revised manuscript, the authors submitted the manuscript for a language service in order to improve it English proficiency and the revised manuscript has been edited by the American Journal Experts service (AJE). Please find the certificate below.

**Comments** **2**: Introduction (line 93-94): “Phytochemical composition may be uncertain due to environmental factors”. What authors would like to convey by making such statements in the introduction? This sentence is presented out of nowhere in introduction.

**Response:** We are sorry about this mistake. Authors agree with the reviewer. The sentence has been deleted.

**Comments** **3**: Line 101: What are those benefits and limitations of BAR-HRM, and what are the benefits of using integrative approach for identification of plants?

**Response:** BAR-HRM analysis has benefits and limitations. The authors added more information about benefits and limitations of BAR-HRM in line 100-108. The benefit of using integrative approach for identification of plants is added in the line 102-105.

**Revised sentences:**

**Line 100-108** **of the revised manuscript:** “Bar-HRM, a sequencing-free method, detects signal alterations during the dissociation of double-stranded DNA generated from the PCR into single-stranded DNA. Each plant species can be differentiated by their individual melting temperature (T_m_), which is correlated to their nucleotide sequences in the target region [23]. Bar-HRM analysis is a fast, cost-effective and reliable method; moreover, a small amount of sample is required for species identification. However, Bar-HRM primer design is challenging when the target sequence has high variation rates across the target amplicon, and Bar-HRM analysis is limited when low-quality DNA templates are used [24].”

**Line 109-113** **of the revised manuscript:** “As mentioned above, each identification method has advantages and limitations; therefore, an integrative approach is proposed to differentiate substitutions or adulterants of herbal species [19, 25]. Combined phytochemical profiles and DNA information can be applied to prevent the use of incorrect herbal species and support the quality of herbal materials to meet international standards [19].”

**Comments** **4**: The introduction lacks a coherence, and authors failed to showcase why they wanted to use HPTLC and BAR-HRM in their study, why not simple TLC or other chromatographic methods to distinguish the study species.

**Response:** Thank you for bringing this into notice.

In this study, the authors designed to use HPTLC instead of the TLC method as the HPTLC has higher resolution than that of the simple TLC, therefore, the HPTLC was chosen to distinguish phytochemical pattern in this work. Therefore, the author revised the manuscript by adding the advantage of HPTLC in **Line 85-93**. As the introduction part lacks a coherence, the manuscript has been edited to make a coherence within the section by adding more information in the introduction part to explain the reason of using the HPTLC and BAR-HRM method for species identification in this study in **Line 85-93** and **Line 100-108,** respectively.

**Line 85-93 of the revised manuscript:** “Thin-layer chromatography (TLC) and high-performance TLC (HPTLC), which recommended in herbal pharmacopoeias of many countries, are reliable methods for phytochemical constituents examination because the methods require target compound as standard reference [17, 18]. HPTLC, a sophisticated form of TLC, provides good separation efficiency due to higher quality of its separation plate. HPTLC exhibits higher accuracy, reproducibility, and ability to document the results compare to TLC [18]. Therefore, this method has been used for phytochemical profile of herbal species. However, uncertain results may occur by environmental factors which affect the chemical composition of herbal species and biological activities of the substances [19].”

**Line 100-108 of the revised manuscript:** “Bar-HRM, a sequencing free method, detects signal alteration during the dissociation of double-stranded DNA into single stranded DNA. Each plant species can be differentiated by their individual melting temperature (Tm) which correlated to their nucleotide sequences in the target region [23]. Bar-HRM analysis exhibits fast, cost-effective and reliable method, moreover, small amount of sample is required for species identification. However, Bar-HRM primer design is challenging when the target sequence has high variation rates across the target amplicon and the Bar-HRM analysis has limited when low quality of DNA template is used [24].”

**Comments** **5**: Methodology: Authors failed to provide the details of herbarium voucher specimen details for the study species?

**Response:** In the previous manuscript version, the authors used the “Code” to present herbarium voucher specimen in Table 1. Therefore, in this revised manuscript, the authors change the column “Code” to “Voucher number”.

**Comments** **6**: Methodology: Also, authors state that fresh leaves were collected, however, they used stem samples for HPTLC analysis? Therefore, authors need to specify the source of such stem samples used in HPTLC.

**Response:** Thank you reviewer for bringing into notice. In this study, stems were used for HPTLC analysis. Therefore, the revised version of manuscript was edited in **Line 121-123** to specify source of sample used in HPTLC experiment. The authors also revised the Method part (HPTLC profiles) and provided detail of sample (**Line 156-157** in revised version) used in HPTLC analysis as following.

**Original sentence**:

**Line 110-111 of the original manuscript:** “Fresh leaves of *A. cocculus* (n=8), *C. caudatus* (n=8) and *M. repandus* (n=8) were collected from various locations across Thailand for the DNA barcoding experiment (Table 1).”

**Revised sentence:**

**Line 121-123 of the revised manuscript:** “Fresh leaves and stems of *A. cocculus* (n=8), *C. caudatus* (n=8) and *M. repandus* (n=8) were collected from various locations across Thailand (Table 1). These collections are legally permitted.”

**Original sentence**:

**Line 142-143 of the original manuscript:** “To obtain the phytochemical profiles of *A. cocculus*, *C. caudatus* and *M. repandus*, 1 g of dried stems from each species were crushed into a fine powder.”

**Revised sentence:**

**Line 156-157 of the revised manuscript:** “To obtain the phytochemical profiles of selected samples, including *A. cocculus* (SS-628), *C. caudatus* (SS-537) and *M. repandus* (SS-583),…”

**Comments** **7**: Line 143: Crushed? How did the authors crushed the dried stem samples?

**Response:** We are sorry for missing information. The author crushed the dried stem samples using a grinder. The sentence has been revised in line 157-158.

**Original sentence:**

**Line 142-143 of the original manuscript:** “…,1 g of dried stems from each species were crushed into a fine powder.”

**Revised sentence:**

**Line 157-158 of the revised manuscript:** “…,1 g of dried stems from each species was crushed into a fine powder using a M 20 Universal mill grinder (IKA, Germany).”

**Comments** **8**: Line 147: sprayed or spotted?

**Response:** Sorry for the mistake. The sentence has been edited by changing “sprayed to spotted” in **Line 162-163**.

**Original sentence**:

**Line 147** **of the original manuscript:** “Then, 5 µl of the extracted solution was sprayed onto an HPTLC plate…”

**Revised sentence:**

**Line 162-163 of the revised manuscript:** “Then, 5 µl of the extracted solution was spotted onto an HPTLC plate...”

**Comments** **9**: Line 150: Authors state that the track distance is 11.4 mm, however they did not indicate whether it is from lower edge of the plate or from the sample spot?

**Response:** Sorry for missing information. The sentence has been revised by adding details of the HPTLC plate setting in **Line 164-166** of the revised manuscript.

**Original manuscript**:

**Line 149-150 of the original manuscript:** “Each individual band was 8 mm in length, the distance between tracks was 2 mm, and the track distance was 11.4 mm.”

**Revised manuscript:**

**Line 164-166 of the revised manuscript:** “Each individual band was 8 mm in length, the distance between tracks was 2 mm, and the track distance was 11.4 mm from the lower edge of the plate.”

**Comments** **10**: Table 2: It is confusing to see these primer pairs and sequence in table 2. Authors indicate in few primers as “This study”, then what is the purpose of providing other primer pair details in table 2? Also, ““This study”, is not indicated in ITS and *psb*A-*trn*H primer pairs. Authors should clarify and include only the primer pairs used in the study, also if they used more than one primer pair to amplify one particular barcode region, it should be included in discussion part.

**Response:** Primers listed in Table 2 are the amplification and sequencing primers. The “This study” terms refer to primers that are originally designed in this work. For those primers without the term “This study” refer to primers that are previously reported. This work, the DNA barcode regions; ITS and *psb*A-*trn*H intergenic spacers regions, were amplifiable using the published ITS primers (ITS1 and ITS4) and *psb*A-*trn*H primers (*psb*A-*trn*HF and *psb*A-*trn*HR), respectively. However, *mat*K region required more primers to complete the full length of nucleotide sequences for *Mallotus repandus* and *Croton caudatus*.

As suggested by the reviewer, the authors agreed to reorganize the Table 2 and the additional primers for *mat*K region of *Mallotus repandus* and *Croton caudatus* have been moved to the Appendix S1 as following.

**Original Table 2.**

**Table 2.** Primers for DNA barcode generation and Bar-HRM analysis.

| **Barcode region** | **Primer name** | **Primer sequence (5′-3′)** | **References** |
| --- | --- | --- | --- |
| **DNA barcode generation** | |  |  |
| *rbc*L | *rbc*L_aF | ATGTCACCACAAACAGAGACTAAAGC | Levin et al., 2003 |
|  | *rbc*L_aR | GTAAAATCAAGTCCACCR(G/A)CG | Kress & Erickson, 2009 |
|  | *rbc*L-Uro_569F | ATGAATGTCTTCGCGGTGGAC | This study |
|  | *rbc*L-R23 | TTTTAGTAAAAGATTGGGCCG | Ohi-Toma et al., 2006 |
| *mat*K | *mat*K-390F | CGATCTATTCATTCAATATTTC | Cuénoud et al. 2002 |
|  | *trn*K72F | TCCGAGGTATCTACTCTTTT | This study |
|  | *mat*K52F | GGGAGGATGGAAATCCGTTG | This study |
|  | *mat*K701F | CGCTATTGGGTGAAAGATCCCT | This study |
|  | *trn*K-3914F | TGGGTTGCTAACTCAATGG | Johnson et al., 1994 |
|  | *mat*K-aF | CTATATCCACTTATCTTTCAGGAGT | Kato et al., 1999 |
|  | *mat*K-MALPR1 | ACAAGAAAGTCGAAGTAT | Dunning & Savolainen, 2010 |
|  | *trn*K1895R | CGCTCAATCCTTACTCGTAT | This study |
|  | *mat*K1253R | GCATTTGACTCCGTACCAACA | This study |
|  | *mat*K1920R | CCCAGTTCCCTTCCTAGACG | This study |
|  | *mat*K-8R | AAAGTTCTAGCACAAGAAAGTCGA | Kato et al., 1999 |
|  | *trn*K-2R | AACTAGTCGGATGGAGTAG | Johnson et al., 1994 |
| *trn*H-*psb*A | *psb*A_*trn*HF | GTTATGCATGAACGTAATGCTC | Sang et al., 1997 |
|  | *psb*A-*trn*HR | CGCGCATGGTGGATTCACAATC | Sang et al., 1997 |
| ITS | ITS1 | TCCGTAGGTGAACCTGCGG | White et al., 1990 |
|  | ITS4 | TCCTCCGCTTATTGATATGC | White et al., 1990 |
|  | ITS5 | GGAAGTAAAAGTCGTAACAAGG | Bellemain et al., 2010 |
| **Bar-HRM analysis** | |  |  |
| *rbc*L | KK-rbcL-HRM-F | TTTCACTCAAGATTGGGTCTCT | This study |
|  | KK-rbcL-HRM-R | TCATCTCCAAAGATCTCGGTCA | This study |

**Revised Table 2:**

**Table 2.** Primers for DNA barcode generation and Bar-HRM analysis.

| **Barcode region** | **Primer name** | | **Primer sequence (5′-3′)** | **References** |
| --- | --- | --- | --- | --- |
| **DNA barcode generation** | | |  |  |
| *rbc*L | *rbc*L_aF | | ATGTCACCACAAACAGAGACTAAAGC | Levin et al., 2003 |
|  | *rbc*L-R23 | | TTTTAGTAAAAGATTGGGCCG | Ohi-Toma et al., 2006 |
| *mat*K | *trn*K-3914F | | TGGGTTGCTAACTCAATGG | Johnson et al., 1994 |
|  | *trn*K-2R | | AACTAGTCGGATGGAGTAG | Johnson et al., 1994 |
| *trn*H-*psb*A | *psb*A_*trn*HF | | GTTATGCATGAACGTAATGCTC | Sang et al., 1997 |
|  | *psb*A-*trn*HR | | CGCGCATGGTGGATTCACAATC | Sang et al., 1997 |
| ITS | ITS1 | | TCCGTAGGTGAACCTGCGG | White et al., 1990 |
|  | ITS4 | | TCCTCCGCTTATTGATATGC | White et al., 1990 |
| **Bar-HRM primers** | |  |  |  |
| *rbc*L | KK-rbcL-HRM-F | | TTTCACTCAAGATTGGGTCTCT | This study |
|  | KK-rbcL-HRM-R | | TCATCTCCAAAGATCTCGGTCA | This study |

**Appendix S1.** Additional primers used for DNA barcode generation used in this study.

| **Species** | **Barcode region** | **Primer name** | **Primer sequence (5′-3′)** |
| --- | --- | --- | --- |
| *Mallotus repandus* | *mat*K | *mat*K52F | GGGAGGATGGAAATCCGTTG |
|  |  | *mat*K1253R | GCATTTGACTCCGTACCAACA |
|  |  | *mat*K701F | CGCTATTGGGTGAAAGATCCCT |
|  |  | *mat*K1920R | CCCAGTTCCCTTCCTAGACG |
| *Croton caudatus* | *mat*K | *mat*K-390F | CGATCTATTCATTCAATATTTC |
|  |  | *mat*K-MALPR1 | ACAAGAAAGTCGAAGTAT |

**Comments** **11**: Authors failed to provide the details of primer design and the tools used to design for the BAR-HRM primer “KK-rbcL-HRM-F” and “KK-rbcL-HRM-R”

**Response:** Thank you for raising to this point. The authors used the MUSCLE program was used for DNA barcode sequences alignment followed by Primer 3 and BLAST software for Bar-HRM primers design.

The manuscript has been revised by adding details of primer design including tools which used for the BAR-HRM primer “KK-rbcL-HRM-F” and “KK-rbcL-HRM-R” design. The information has been added in **line 210-212** and **line 214-217**.

**Line 210-212 of the revised manuscript:** “To design Bar-HRM primers, nucleotide sequences obtained from the four DNA barcode regions of *M. repandus, A. cocculus* and *C. caudatus* were aligned by MUSCLE with gap open = –400; gap extend = 0; clustering method = UPGMB; and Min Diag Length = 24.”

**Line 214-217 of the revised manuscript:** “Primer 3 and BLAST software were used for primer design. The Bar-HRM forward (KK-rbcL-HRM-F) and reverse primers (KK-rbcL-HRM-R) were designed based on the conserved regions of the *rbc*L gene of the three plants. The targeted amplicon provided a 102 bp amplicon with 9 polymorphic sites of the *rbc*L gene.”

**Comments** **12**: Authors can really shorten the DNA extraction part and PCR amplification part in the methodology section.

**Response:** Thank you reviewer for your suggestion. The manuscript has been revised by shorten the DNA extraction part and PCR amplification part in **Line 175-184 of the revised manuscript**.

**Original paragraph of DNA extraction part:**

**Line 159-173 of the original manuscript:** “Fresh leaves of authentic *A. cocculus*, *C. caudatus* and *M. repandus* were ground into a fine powder with liquid nitrogen. Genomic DNA was extracted from 50 mg of fine powder using a DNeasy Plant Mini Kit (Qiagen, Germany) following the manufacturer’s instructions. A GENECLEAN Kit (MP Biomedicals, USA) was used to purify the genomic DNA according to the manufacturer’s protocol. The quantity of the extracted DNA was determined spectrophotometrically using a NanoDrop One UV-Vis Spectrophotometer (Thermo Scientific, USA). DNA quality was observed by agarose gel electrophoresis. Genomic DNA was run on 0.8% (w/v) agarose in 1X TAE gel containing 1X RedSafe nucleic acid staining solution (iNtRON Biotechnology, USA) at 100 V for 30 min. The agarose gel was analyzed with a UVP GelSolo (Analytik Jena GmbH, Germany) gel documentation system. Images were taken by onboard VisionWorks software (Analytik Jena GmbH, Germany). Genomic DNA was stored at -20 ℃ for further use. Genomic DNA was extracted and purified from the purchased crude drugs called “Kho-Khlan”, mixed powder of plants and laboratory-made YPSKK formulae using the methods described above. Genomic DNA quantification and qualification were conducted as described for the authentic plant samples.”

**Revised paragraph of DNA extraction part:**

**Line 175-184 of the revised manuscript:** “Genomic DNA from leaves of the samples, the purchased crude drugs, mixed herbal powder and laboratory-made YPSKK formulae were extracted using a DNeasy Plant Mini Kit (Qiagen, Germany) and further purified using a GENECLEAN Kit (MP Biomedicals, USA) according to the manufacturer’s protocol. DNA quantity and quality were determined using a NanoDrop One UV–Vis Spectrophotometer (Thermo Scientific, USA) and agarose gel electrophoresis, respectively. Genomic DNA was run on 0.8% (w/v) agarose in 1X TAE gel containing 1X RedSafe nucleic acid staining solution (iNtRON Biotechnology, USA) at 100 V for 30 min. Agarose gel was analyzed with a UVP GelSolo (Analytik Jena GmbH, Germany) gel documentation system, and images were taken by onboard VisionWorks software (Analytik Jena GmbH, Germany). Genomic DNA was stored at -20℃ for further use.”

**Comments** **13**: One of my concern with the DNA barcoding and BAR-HRM in this study is, authors have not used any closely related plant groups as control or to highlight that BAR-HRM can distinguish closely related species. It is obvious and no surprise that the study plants belong to Euphorbiaceae and Menispermaceae which shows enough genetic variation to be distinguished. In fact, experienced filed botanist and microscopy analysis can easily distinguish these study species, why anyone wants to use BAR-HRM to distinguish plants that belong to different families and has enough morphological variation itself.

**Response:** Thank you reviewer for your comment. We totally appreciate your concern. The reason that the authors have not used any closely related species in this study because the plants called “Kho-Khlan”, in fact, they are only three herbal species; *A. cocculus, C. caudatus* and *M. repandus* found in Thai herbal markets. Therefore, we have not used other plants in this study.

In the herbal markets, normally the herbal materials are sold in the processed forms such as small pieces of stem and powder which its identities have been lost. This is challenging us to identify small pieces of stem or herbal powder by morphological analysis or microscopic examination. Bar-HRM analysis will benefit people who involved with regulatory policy of herbal products and herbal industry as the Bar-HRM analysis supports species identification of highly processed raw materials. The Bar-HRM analysis also requires low amount of DNA sample and the method is able to amplify fragmented DNA, which normally found in the DNA of highly processed materials.

**Comments** **14**: Another concern with HPTLC is, why authors have not derivatized the HPTLC plate using any derivatizing reagents?, without derivatization, and using only 366 nm, only UV active compounds are visible and the data provided by authors are not sufficient to claim that the authors developed a HPTLC method.

**Response:** We appreciate the reviewers’ comments. The reason why the authors did not derivatize the HPTLC plate because the species-specific bands of the *Mallotus repandus*, *Anamirta cocculus* and *Croton caudatus* were simply detected under the wavelength of 366 nm plus the high quality of HPTLC plate that provides good separation efficiency, therefore, no further derivatizing reagents is needed in this study. Moreover, the authors agree that HPTLC method we adapt in this study are not sufficient to claim that the authors develop a HPTLC method. Therefore, the term “develop HPTLC” has been change to “HPTLC”.

**Reviewer #2:**

The present study established a combined Bar-HRM and HPTLC technique to identify the correct species, *Mallotus repandus*, from authentic *Anamirta cocculus* and *Croton caudatus*. This method was successfully applied to identify crude drugs and multiherbal mixed formulae and serve as a quality control tool for preventing accidental confusion of herbal species sharing the same common name. The experiment design is logical and fine. However, there are still some issues that merit authors’ attention.

**Comments** **1**: The writing of the “Introduction” is too wordy, especially lines 59-76. Simply explain that *A. cocculus* and *C. caudatus* can't be used as substitutes of *M. repandus* in clinical practices.

**Response:** We appreciate the reviewers’ comments. The authors agree with the reviewer that the introduction is too wordy. Therefore, the authors rewrite the paragraph to be more concise.

Although the *A. cocculus* and *C. caudatus* cannot be used as substitutes of *M. repandus* in clinical practices but the *A. cocculus* and *C. caudatus* also possess their own medicinal properties. Therefore, the authors would like to use the first paragraph in the Introduction part to describe their healing properties to mention that they cannot be used because of the difference in healing properties and the paragraph has been revised in line 57-74 as following.

**Original sentence**:

**Line 59-76 of the original manuscript:** “However, at least three herbs, namely, *Mallotus repandus* (Willd.) Müll. Arg. (Euphorbiaceae), *Croton caudatus* Gleiseler (Euphorbiaceae) and *Anamirta cocculus* (L.) Wight & Arn (Menispermaceae), share the common name “Kho-Khlan”, and they have different healing properties [4] (Fig 1). *M. repandus* is the only official “Kho-Khlan” species and is prescribed as a main component in the formula. *M. repandus* has long been used for the relief of muscle pain [2]. *C. caudatus* is administered for headaches, visceral pain, and rheumatism [5]. This species is also reported to treat malaria, fever, numbness, and constipation [6]. In some parts of Asia, *C. caudatus* is applied as a poultice to treat fever and sprains [7]. In addition, the crude extract of *C. caudatus* seeds can protect against larvae of mosquitoes [8]. The folk literature indicates that most of the plants in the genus *Croton* cause irritation and allergic responses [9]. *A. cocculus* is used in the treatment of blood stasis and fever and stimulates the central nervous system [11]. This species is recorded as a restorative medical herb in the southern region of Thailand [11]. The plant, however, contains very strong neurotoxin compounds, such as picrotoxin, picrotin, methyl picrotoxate, dihydroxypicrotoxinin, picrotoxic acid and a sesquiterpene mixture of picrotoxinin, that affect the central nervous system (CNS) of vertebrates [12-14]. Seeds of *A. cocculus* are used to eliminate unwanted wild fish in aquaculture ponds and to kill birds [15]. A case study reported that consuming *A. cocculus* berries caused extensive brain hemorrhage in cattle [14]. Small amounts of *A. cocculus* are highly toxic and fatal if consumed by humans [12]. Although the substances in *A. cocculus* are harmful, the herb is still utilized in Thai traditional medicine due to the belief that a very small dose of toxic substances can be neutralized by other compounds in the herbal formula [16]”

**Revised sentence:**

**Line 57-74 of the revised manuscript:** “*M. repandus* (Euphorbiaceae) shares the common name “Kho-Khlan” with *Croton caudatus* Gleiseler (Euphorbiaceae) and *Anamirta cocculus* (L.) Wight & Arn (Menispermaceae) (Fig. 1). However, only *M. repandus* (Fig. 1A) is an official plant species in NLEM.

The stem of *M. repandus* has long been used for the relief of muscle pain in Thai traditional medicine [2]. *C. caudatus* is administered for headaches, visceral pain, rheumatism, fever, and constipation [4-6]. The crude extract of *C. caudatus* seeds can protect against mosquito larvae [7]. *A. cocculus* is used in the treatment of blood stasis and fever, stimulates the central nervous system [8] and is recorded as a restorative medical herb in the southern region of Thailand [9]. However, a previous report showed that *C. caudatus* causes irritation and allergic responses [10], while *A. cocculus* contains very strong neurotoxin compounds that affect the central nervous system (CNS) of vertebrates, such as picrotoxin, picrotin, methyl picrotoxate, dihydroxypicrotoxinin, picrotoxic acid and a sesquiterpene mixture of picrotoxinin [11-13]. Seeds of *A. cocculus* are also used to eliminate unwanted wild fish in aquaculture ponds and to kill birds [14]. Consuming *A. cocculus* berries causes extensive brain hemorrhage in cattle, while small amounts of *A. cocculus* are highly toxic and fatal if consumed by humans [11, 13]. Although the substances in *A. cocculus* are harmful, the herb is still utilized in Thai traditional medicine due to the belief that a very small dose of toxic substances can be neutralized by other compounds in the herbal formula [15].”

**Comments** **2**: The description of the existing identification methods in the second paragraph of “Introduction” is logically confused. Please rewrite.

**Response:** Thank you reviewer for raising this point. The Introduction part was rewritten to be more logical. The introduction section has been revised in line 80-117 of the revised manuscript as following.

**Original paragraph:**

**Line 83-106 of the original manuscript:** “Only *M. repandus* is used for YPSKK preparation, and it is challenging to differentiate among the three “Kho-Khlan” species (Fig 1A) when they are in processed forms (Fig 1B-C). Thus, a precise identification tool is necessary to avoid negative health effects that can occur by using raw materials from incorrect species. Classical procedures for the identification of herbs involve organoleptic methods and micro- and macroscopic and chemical characters [17]. The organoleptic and micro- and macroscopic methods are basic techniques that require simple equipment and experienced personnel for taxonomic examination. Thin-layer chromatography (TLC) is used for phytochemical identification of raw herbal material and is also recommended in the Thai Herbal Pharmacopoeia [18]. TLC and other tools, such as high-performance TLC (HPTLC) and high-performance liquid chromatography (HPLC), require standard compounds as references. Phytochemical composition may be uncertain due to environmental factors. Although genetic methods based on DNA sequence analysis require specialists and are cost effective, these methods provide species-level information, and a small number of samples is needed for the identification process [19]. For a decade, DNA barcoding has been established and applied for species authentication and identification. DNA barcoding coupled with high-resolution melting (Bar-HRM) analysis have gained attention for its fast identification of herbal species such as *Vaccinium myrtillus* [20], *Mitragyna speciosa* [21] and *Ardisia gigantifolia* [22]. However, Bar-HRM methods have different benefits and limitations; therefore, an integrative approach is proposed to differentiate substitutions and adulterants of herbal species [19, 23]. In this study, we aimed to utilize phytochemical profiles and DNA information to differentiate *M. repandus* from *C. caudatus* and *A. cocculus*. HPTLC and Bar-HRM approaches were combined to create a simple and fast identification method for the quality control of “Kho-Khlan” raw material in the herbal industry.”

**Revised** **paragraph:**

**Line 80-117 of the revised manuscript:** “The stem of *M. repandus* is used for the YPSKK formula. Crude drugs of *M. repandus* are commercially provided in both powdered form and small pieces of stem, which are challenging for species differentiation (Fig. 1B-C). Although raw materials of herbal medicine can be examined by simple organoleptic methods and macroscopic and microscopic methods, experienced personnel for taxonomic examination are required [16]. Thin-layer chromatography (TLC) and high-performance TLC (HPTLC), which are recommended in the herbal pharmacopoeias of many countries, including Thailand, are reliable methods for phytochemical constituent examination; however, the methods require a target compound as a standard reference [17, 18]. HPTLC, a sophisticated form of TLC, provides good separation efficiency due to the higher quality of its separation plate. HPTLC exhibits higher accuracy, reproducibility, and ability to document the results than TLC [18]. Therefore, this method has been used to determine the phytochemical profile of herbal species. However, uncertain results may occur due to environmental factors that affect the chemical composition of herbal species and biological activities of the substances [19]. In recent years, a molecular approach called the DNA barcoding technique has gained demand in species identification because it is an accurate, cost-effective and reliable tool for species identification. The DNA barcoding method provides species-level information, and small amounts of samples are needed for the identification process [19].

Currently, DNA barcoding coupled with high-resolution melting (Bar-HRM) analysis has gained attention for its rapid identification of herbal species such as *Vaccinium myrtillus* L. [20], *Mitragyna speciosa* Korth [21] and *Ardisia gigantifolia* Stapf [22]. Bar-HRM, a sequencing-free method, detects signal alterations during the dissociation of double-stranded DNA generated from the PCR into single-stranded DNA. Each plant species can be differentiated by their individual melting temperature (T_m_), which is correlated to their nucleotide sequences in the target region [23]. Bar-HRM analysis is a fast, cost-effective and reliable method; moreover, a small amount of sample is required for species identification. However, Bar-HRM primer design is challenging when the target sequence has high variation rates across the target amplicon, and Bar-HRM analysis is limited when low-quality DNA templates are used [24].

As mentioned above, each identification method has advantages and limitations; therefore, an integrative approach is proposed to differentiate substitutions or adulterants of herbal species [19, 25]. Combined phytochemical profiles and DNA information can be applied to prevent the use of incorrect herbal species and support the quality of herbal materials to meet international standards [19]. In this study, we aimed to utilize HPTLC and Bar-HRM analysis to differentiate a pain relief herb, *M. repandus*, from *C. caudatus* and *A. cocculus*, which share the common name Kho-Khlan. Combined approaches were used to create a simple and rapid identification method for the quality control of the Kho-Khlan raw material in the herbal industry.”

**Comments** **3:** Why did authors select the *rbc*L region for Bar-HRM analysis for the differentiation of *M. repandus* from *A. cocculus* and *C. caudatus*?

**Response:** The authors selected *rbc*L gene as a target region for Bar-HRM analysis due to the

sequence alignment result of the DNA barcode regions among *M. repandus*, *A. cocculus* and *C. caudatus*, we found the *rbc*L region possess a target site of 102 bp which contained two conserve regions and one variable region. The 102 bp amplicon has one conserve region at the 5´- and another at the 3´-end. Nine nucleotide sites (within the variable region) flanked the two conserve regions and results in the difference of melting temperature among each 102 bp amplicons of *M. repandus*, *A. cocculus* and *C. caudatus*. These characters, two conserve regions flank by nucleotide variable sites and the amplicon site less than 300 bp, suite for Bar-HRM primer design and only *rbc*l gene exhibits the characters.

Therefore, the authors added the reason for the selection of the *rbc*L region for Bar-HRM analysis to differentiate *M. repandus* from *A. cocculus* and *C. caudatus* in line 389-396 and line 400-403 as following.

**Line 389-396 of the revised manuscript:** “Since the gene sequences in the *rbc*L regions of *A. cocculus, C. caudatus* and *M. repandus* possess two conserved sites flanking nine nucleotide polymorphism sites, this region is suitable for the design of Bar-HRM primers. The *rbc*L region was chosen as a targeted amplified region for Bar-HRM analysis. The nucleotide variation within 102 bp of PCR amplicons amplified from the three species resulted in different melting temperatures when analyzed by the Bar-HRM approach. An amplicon of 102 bp is in the range of desired amplicon lengths for the Bar-HRM analysis (<300 bp) suggested by Osathanunkul et al., 2015 [25].”

**Line 400-403 of the revised manuscript:** “Moreover, the use of the *rbc*L region for species differentiation at the genus level has been revealed [38]. These results support our conclusion on the reliability of the *rbc*L region as a potential DNA barcode marker for discrimination of the nonrelated species that belong to different genera, *A. cocculus, C. caudatus* and *M. repandus*.”

**Comments** **4:** I cannot find the HPTLC bands of *C. caudatus* (Rf = 0.55) in tracks 6-7 in figure 3. Besides, the enlarged image on the right in Figure 3 should have a scale.

**Response:** Thank you for raising this point. The authors agreed with reviewer that HPTLC bands of *C. caudatus* (Rf = 0.55) in tracks 6-7 in figure 3 are missing. In order to see the missing band, the authors adjusted mobile phase ratio from toluene:acetone:formic acid, 5:3:0.5 (v/v/v) to 5:4:0.5 (v/v/v). This change resulted in the Rf alteration of species-specific band from Rf=0.55 to Rf=0.60 (see the revised Fig. 2). Therefore, the authors changed the original Fig. 3 with the revised Fig. 3. Scale in revised Fig. 3 has been added in the enlarge image revised Fig. 3 as shown below.

**Original Fig 2.** **High-performance thin-layer chromatography (HPTLC) chromatogram of ethanolic extracts under UV 366 nm.** Track 1: *A. cocculus*, track 2: *C. caudatus*, track 3: *M. repandus*, tracks 4–9: crude drugs CD1-CD6. A toluene:acetone:formic acid mixture (5:3:0.5, v/v/v) was used as the mobile phase. White arrows indicate the characteristic bands of each authentic plant species.

**Revised Fig. 2.** **High-performance thin-layer chromatography (HPTLC) chromatogram of ethanolic extracts under UV at 366 nm.** Track 1: *A. cocculus*, track 2: *C. caudatus*, track 3: *M. repandus*, tracks 4–9: crude drugs CD1-CD6. A toluene:acetone:formic acid mixture (5:4:0.5, v/v/v) was used as the mobile phase. White arrows indicate the characteristic bands of each authentic plant species.

**Original Fig 3. HPTLC chromatograms of ethanolic extracts of authentic species and four laboratory-made YPSKK formulae under UV 366 nm.** Track 1: *A. cocculus*, track 2: *C. caudatus*, track 3: *M. repandus*, tracks 4–5: F-A, tracks 6–7: F-C, tracks 8–9: F-M and tracks 10–11: F-ACM. A toluene:acetone:formic acid (5:3:0.5, v/v/v) mixture was used as the mobile phase. White arrows indicate the characteristic bands of each plant species.

**Revised Fig** **3. HPTLC chromatograms of ethanolic extracts of authentic species and four laboratory-made YPSKK formulae under UV at 366 nm.** Track 1: *A. cocculus*, track 2: *C. caudatus*, track 3: *M. repandus*, tracks 4–5: F-A, tracks 6–7: F-C, tracks 8–9: F-M and tracks 10–11: F-ACM. A toluene:acetone:formic acid (5:4:0.5, v/v/v) mixture was used as the mobile phase. White arrows indicate the characteristic bands of each plant species.

**Comments** **5:** This study showed that compared with *A. cocculus*, species *C. caudatus* and *M. repandus* were more difficult to distinguish. It is suggested that authors add some discussion to elaborate on how the two methods, Bar-HRM and HPTLC, complement each other to distinguish the three species, especially the distinction between *C. caudatus* and *M. repandus*.

**Response:** Thank you for reviewer’s suggestion. The authors added information in the discussion section to elaborate on how the two methods, Bar-HRM and HPTLC, complement each other to distinguish the three species in the discussion part in line 411-414 as following.

**Line 411-414** **of the revised manuscript:** “This suggested that Bar-HRM and HPTLC can complement each other to distinguish *C. caudatus* and *M. repandus* when uncertainty in phytochemical constituents is observed. Bar-HRM analysis using genetic information can be used to clarify the ambiguous result, as the genetic information is stable.”

*****************************
